# New Stilbene Dimer Compounds from the Fruits of *Hypericum patulum* and Their Antioxidant and Anti-α-Glucosidase Activities

**DOI:** 10.3390/molecules31173072

**Published:** 2026-09-01

**Authors:** Yang Wang, Qian-Lian Yin, Li Jiang, Shi-Hai Zhang, Xue Ma, Jia Sun, Yong-Jun Li

**Affiliations:** 1 State Key Laboratory of Discovery and Utilization of Functional Components in Traditional Chinese Medicine, Engineering Research Center for the Development and Application of Ethnic Medicine and TCM (Ministry of Education), Guizhou Provincial Engineering Research Center for the Development and Application of Ethnic Medicine and TCM, Guizhou Medical University, Guian New Area, Guiyang 561113, China; 15180803607@163.com (Y.W.); 17886278417@163.com (Q.-L.Y.); 15585155697@163.com (L.J.); zhangshihai@gmc.edu.cn (S.-H.Z.); xuema0111@163.com (X.M.); 2 School of Pharmacy, Guizhou Medical University, Guian New Area, Guiyang 561113, China

**Keywords:** *Hypericum patulum* Thunb., Miao medicine, stilbene dimers, enantiomers, anti-*α*-glucosidase, antioxidant

## Abstract

Three previously undescribed phenolic dimers, including a pair of enantiomeric stilbene dimers (**1a**/**1b**) and one optically pure stilbene dimer (**2**), were separated from the fruits of the Miao medicine *Hypericum patulum* Thunb. Structural clarification and assignment of absolute configurations were achieved through 1D/2D NMR spectroscopy, optical rotation measurements, and analysis of experimental electronic circular dichroism (ECD) spectra. Their antioxidant and anti-*α*-glucosidase activities were evaluated. The new stilbene dimers exhibited potent antioxidant activity in both DPPH and ABTS free radical scavenging assays. Moreover, (−)-caesalstilbene C (**1b**) demonstrated the highest anti-DPPH and anti-ABTS activities among all isolated compounds. Compounds **1**–**2** also showed significant *α*-glucosidase-inhibitory activity, comparable to the positive control acarbose. These findings provide a foundation for developing novel antioxidants or α-glucosidase inhibitors as lead compounds in the search for new antidiabetic agents.

## 1. Introduction

The genus *Hypericum* L. comprises a chemically diverse group of medicinal plants widely distributed in temperate and subtropical regions. Phytochemical investigations of *Hypericum* species have revealed numerous structurally diverse secondary metabolites, including polycyclic polyprenylated acylphloroglucinols (PPAPs), xanthones, flavonoids, naphthodianthrones, meroterpenoids, phenolic acids, and other phenolic constituents [1,2,3]. Among these metabolites, PPAPs, xanthones, and naphthodianthrones are considered representative chemical constituents of the genus and contribute substantially to its phytochemical diversity. Compounds isolated from Hypericum species have also exhibited a broad range of experimentally demonstrated biological activities, including anti-inflammatory, antioxidant, antimicrobial, cytotoxic, neuroactive, and enzyme-inhibitory effects [1,2,3,4]. These findings highlight the genus Hypericum as an important source of structurally distinctive and biologically active natural products.

*Hypericum patulum* Thunb. ex Murray is a perennial species distributed mainly in southwestern and central China, as well as Taiwan, China. In Guizhou Province, the whole plant has traditionally been used by the Miao ethnic group for medicinal purposes. In Miao ethnomedical practice, it is used for “clearing heat and detoxifying,” promoting blood circulation, relaxing tendons, soothing the liver, and stopping bleeding [5]. These descriptions represent traditional therapeutic concepts and should be distinguished from pharmacological activities established through modern experimental studies. Previous phytochemical investigations of *H. patulum* have identified several structurally diverse classes of secondary metabolites, including PPAPs and related acylphloroglucinol derivatives [6,7,8,9], meroterpenoids [10], xanthones and xanthone glycosides [11,12,13], flavonoids [14], and other phenolic and glycosidic constituents [15,16]. Some of these constituents have demonstrated cytotoxic, immunosuppressive, anti-inflammatory, wound healing, sodium current-inhibitory, and glucose metabolism-regulating activities in cellular or animal models [6,7,8,9,10,14], indicating that *H. patulum* is a promising source of bioactive secondary metabolites.

Among the natural products occurring in medicinal plants, phenolic dimers constitute a structurally and stereochemically diverse group. They are commonly formed through oxidative coupling of phenolic, stilbenoid, or flavonoid-related precursors and may contain multiple stereogenic centers [17,18]. Certain phenolic dimers and related oligomers have been reported to exhibit antioxidant, antimicrobial, cytotoxic, anti-inflammatory, and carbohydrate-hydrolyzing enzyme-inhibitory activities [17,18,19,20]. Nevertheless, compared with the extensive studies of PPAPs and xanthones in *Hypericum*, phenolic dimers have received considerably less attention, and their distribution, stereochemical diversity, and biological properties within the genus remain insufficiently characterized [21,22].

Previous studies of *H. patulum* have focused predominantly on PPAPs, meroterpenoids, xanthones, flavonoids, and other monomeric or polycyclic metabolites. Although several xanthone glycosides, monoterpenoid glycosides, and other constituents have recently been isolated from its fruits [15,16], phenolic dimers from this plant part remain poorly investigated. From a biosynthetic perspective, the occurrence of such dimers may reflect oxidative coupling pathways involving stilbenoid- or flavonoid-related precursors. Their identification may therefore expand the known metabolic profile of *H. patulum* and provide additional chemical evidence for comparison with related *Hypericum* species. However, further comparative studies are required before these compounds can be regarded as definitive chemotaxonomic markers.

**Figure 1:**
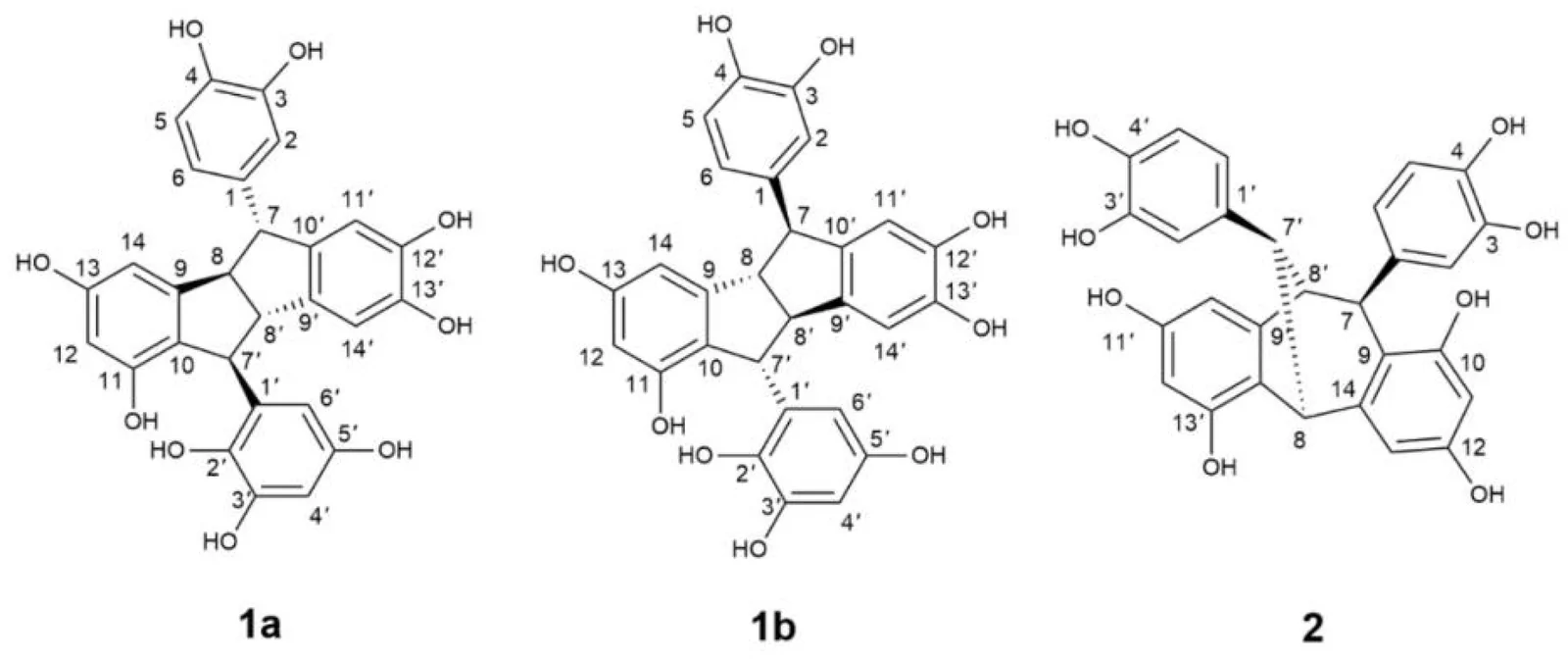
Structures of compounds **1**–**2**.

To address this knowledge gap, we conducted a phytochemical investigation of the fresh fruits of *H. patulum* as part of our continuing search for bioactive natural products from Miao medicinal plants in Guizhou Province. This study resulted in the isolation of three phenolic dimers, namely (+)-caesalstilbene C (**1a**), (−)-caesalstilbene C (**1b**), and (−)-ampelopsin J (**2**) (Figure 1). Their structures and absolute configurations were established through comprehensive spectroscopic analyses and appropriate stereochemical methods. In addition, their antioxidant and α-glucosidase-inhibitory activities were evaluated. The present findings expand the phytochemical profile of *H. patulum*, provide further evidence for the occurrence of structurally diverse phenolic coupling products in the genus Hypericum, and offer a basis for future investigations of their biological and potential chemotaxonomic significance.

## 2. Results

### 2.1. Structure Determination

The 30% ethanol extract of the fresh ripe fruits of *H. patulum* was subjected to repeated column chromatography (CC) using silica gel and Sephadex LH-20, resulting in the isolation of three new phenolic dimers, namely (+)-caesalstilbene C (**1a**), (−)-caesalstilbene C (**1b**), and (−)-ampelopsin J (**2**) (Figure 1).

**Figure 2:**
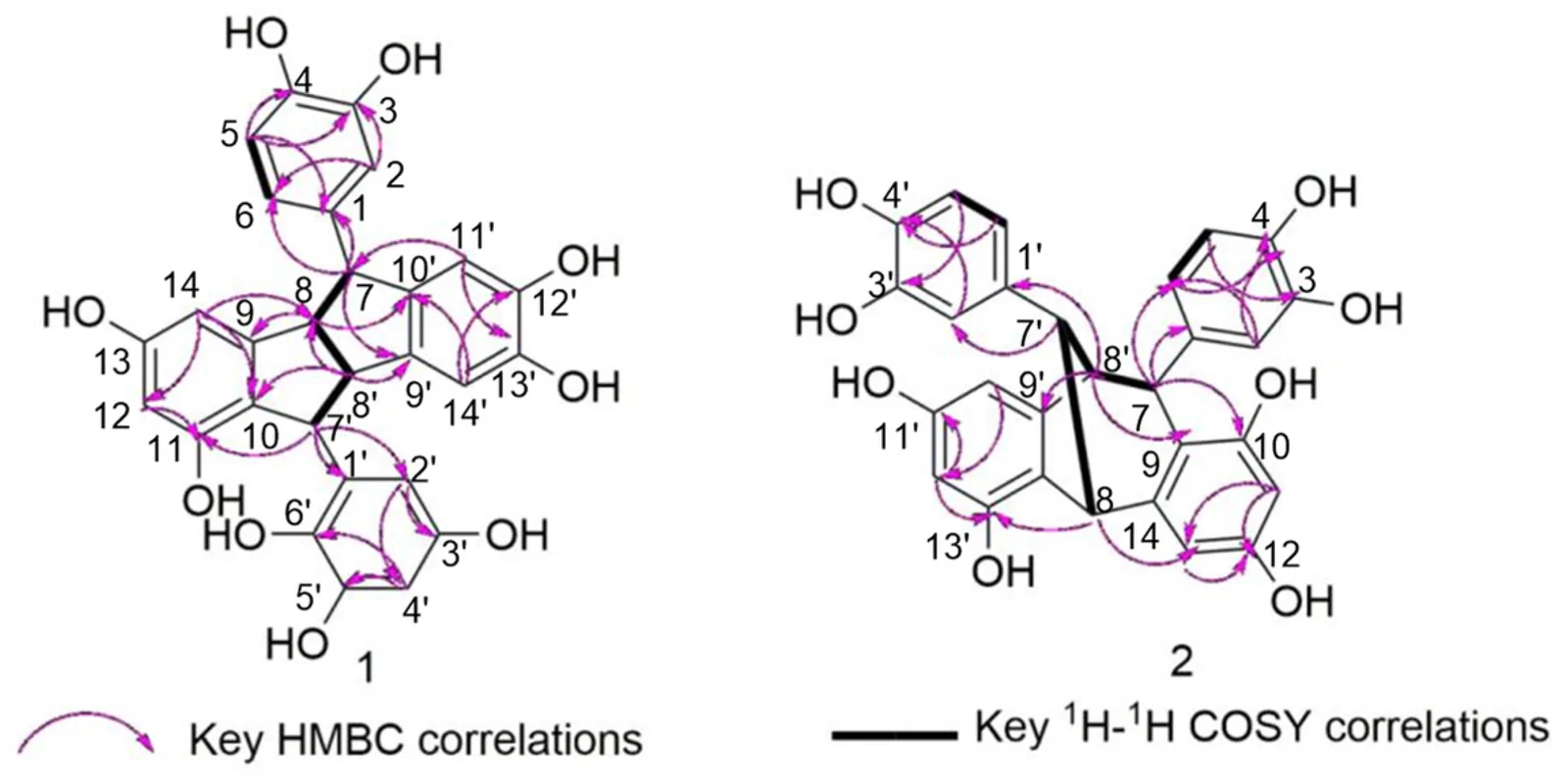
Key ^1^H-^1^H COSY and HMBC correlations of compounds **1**–**2**.

**Figure 3:**
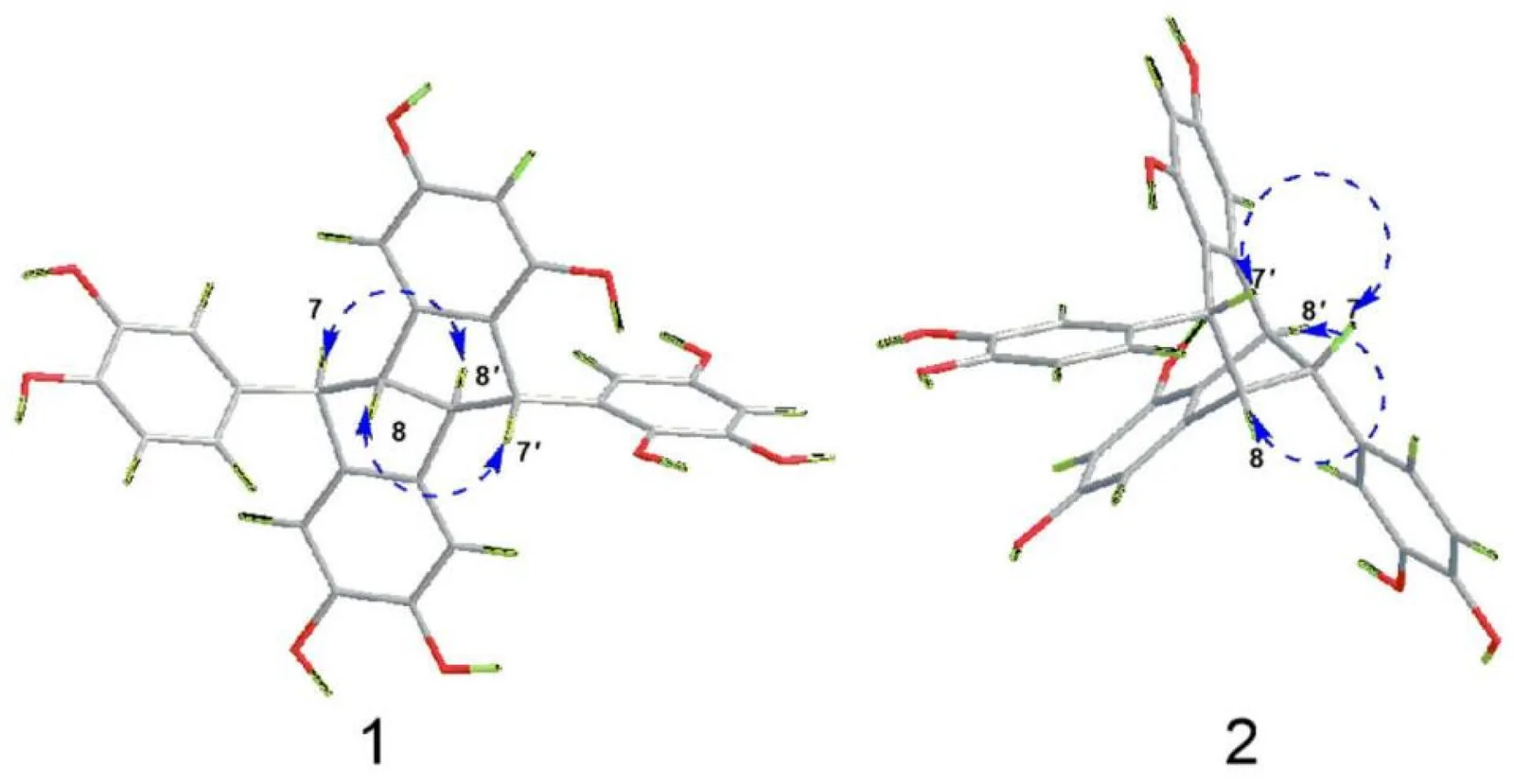
Key NOESY correlations of compounds **1**–**2**.

**Figure 4:**
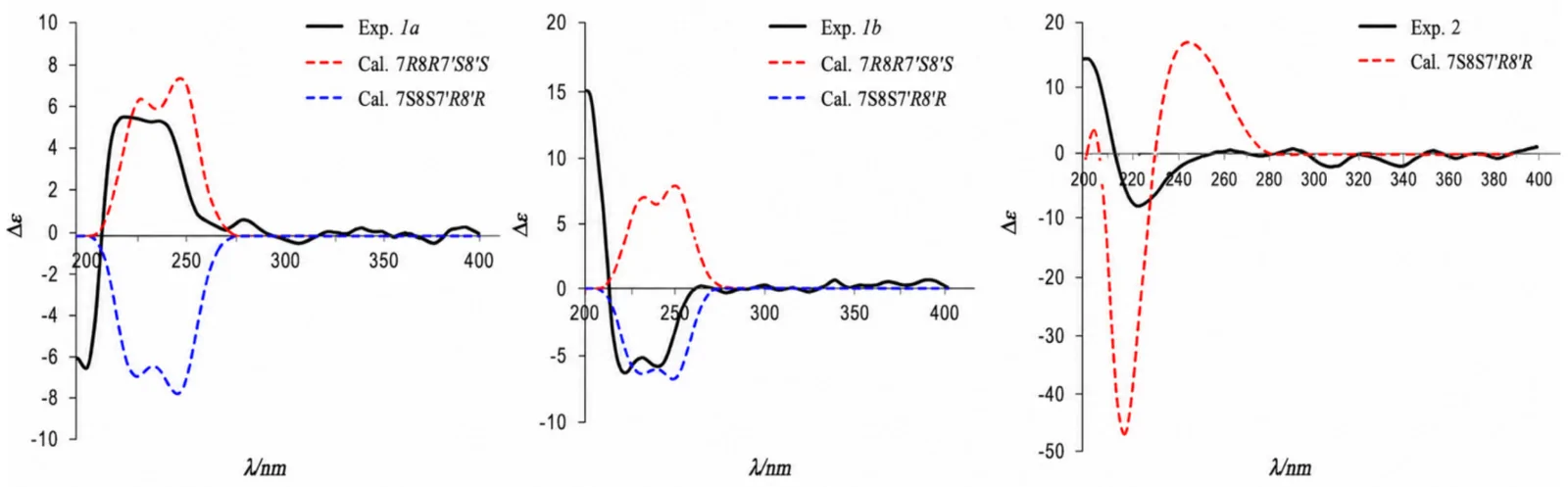
Calculated and experimental ECD spectra of compounds **1**–**2**.

Compound **1** was isolated as a brown solid. Its molecular formula was established as C_28_H_22_O_9_ by HR-ESI-MS (*m*/*z* 501.1160 [M − H]^−^, calcd for C_28_H_21_O_9_^−^ 501.1180). The ^1^H-NMR spectrum shows four methyne signals [*δ*_H_ 4.33 (H-7′), 4.21 (H-7), 3.83 (H-8′) and 3.81 (H-8)]; a 1,2,4-trisubstituted benzene ring [*δ*_H_ 6.68 (H-5), 6.50 (H-6) and 6.49 (H-2)]; two 1,2,3,5-tetrasubstituted benzene rings [*δ*_H_ 6.45 (H-12), 6.12 (H-14), 6.14 (H-6′), and 6.08 (H-4′)]; and a 1,2,4,5-tetrasubstituted benzene ring [*δ*_H_ 6.42 (H-11′) and 6.93 (H-14′)]. The ^13^C-NMR spectrum provides 28 carbon signal peaks, including four methyne carbon signals [*δ*_C_ 61.0 (C-8), 60.7 (C-8′), 58.9 (C-7) and 56.0 (C-7′)], and the rest are carbon signals on the benzene rings. Further analysis of compound **1**’s 2D NMR revealed that it has the similar planar skeletons as Caesalstilbene A [23], with the difference being the additional hydroxyl substitution on the benzene ring’s C-3, 2′. The HMBC (Figure 2) correlations from H-5 and H-2 to C-3, together with the correlation from H-4′ to C-2′, indicated the presence of hydroxy substituents at C-3 and C-2′. Furthermore, based on the HMBC correlations from H-7 to C-1, C-6, and C-9′; from H-8 to C-9 and C-10′; from H-7′ to C-11, C-1′, and C-6′; and from H-8′ to C-9′, C-8, and C-10, compound **1** was determined to possess the same core skeleton as caesalstilbene A. In the NOESY spectrum (Figure 3), correlations were observed between H-7′ and H-8, and between H-7 and H-8′, indicating that H-7 and H-8′ were *β*-oriented, whereas H-7′ and H-8 were *α*-oriented. Compound **1** showed no apparent Cotton effects in the ECD spectrum (Figure 4), and its specific optical rotation was close to zero, suggesting that it was racemic. Subsequently, compound **1** was separated by chiral HPLC to afford a pair of enantiomers, **1a** and **1b**, in a 1:1 ratio. The absolute configurations of **1a** and **1b** were established by comparison of their experimental ECD spectra with the corresponding TDDFT-calculated ECD spectra. To determine their absolute configurations, the ECD spectra of **1a** and **1b** were calculated at the B3LYP-D3(BJ)/6-31G level. The calculated ECD spectra were in good agreement with the corresponding experimental spectra (Figure 4), allowing the absolute configurations of **1a** and **1b** to be assigned as 7R, 8R, 7′S, 8′S and 7S, 8S, 7′R, 8′R, respectively. Therefore, the structures of compounds **1a** and **1b** are identified as (+)-Caesalstilbene C (**1a**) and (−)-Caesalstilbene C (**1b**), and the detailed data assignments are shown in Table 1.

Compound **2** was obtained as a pale yellow solid and was assigned the molecular formula C_28_H_22_O_8_ based on HR-ESI-MS data (*m*/*z* 485.1244 [M − H]^−^, calcd for C_28_H_21_O_8_^−^ 485.1231). The ^1^H NMR spectrum showed signals for four methine protons at *δ*_H_ 4.02 (2H, H-7 and H-8), 3.56 (H-7′), and 3.22 (H-8′). It also exhibited aromatic proton signals attributable to four benzene rings, including two ABX-type spin systems [*δ*_H_ 6.71 (H-5), 6.64 (H-2), and 6.57 (H-6); *δ*_H_ 6.49 (H-5′), 6.33 (H-2′), and 6.23 (H-6′)] and two meta-coupled systems [*δ*_H_ 6.36 (H-10′) and 6.01 (H-12′); *δ*_H_ 6.34 (H-13) and 6.08 (H-11)]. The ^13^C NMR spectrum displayed 28 carbon resonances, among which the signals at *δ*_C_ 59.5 (C-8′), 51.2 (C-7′), 49.6 (C-8), and 47.8 (C-7) were assigned to four methine carbons, while the remaining signals were attributable to aromatic carbons. Further analysis of the 2D NMR data of compound **2** indicated that it possessed the same planar structure as the known compound (−)-ampelopsin F, except that the hydrogen atoms at C-3 and C-3′ in (−)-ampelopsin F were replaced by hydroxy groups in compound **2**. The HMBC spectrum (Figure 2) showed key correlations from H-7 to C-1 and C-6, from H-7′ to C-1 and C-6, from H-8 to C-7′, C-13′, C-14′, C-9, C-13, and C-14, and from H-8′ to C-1, C-9, C-1′, C-9′, and C-14′. These correlations further supported the proposed planar structure of compound **2**. Because the signals of H-7 and H-8 overlapped in the NOESY spectrum, their relative configuration could not be determined from the NOESY data. However, the signals corresponding to the four methine protons [*δ*_H_ 4.02 (2H, H-7 and H-8), 3.56 (H-7′), and 3.22 (H-8′)] exhibited very small coupling constants and appeared as singlets, suggesting that the dihedral angles between the vicinal protons were approximately 90°. Accordingly, H-7 and H-7′ were assigned an α-orientation, whereas H-8 and H-8′ were assigned a *β*-orientation (Figure 3). Comparison with the literature data [24] revealed that the structure of compound **2** was similar to that of (−)-ampelopsin F. According to the literature, H-7, H-8, H-7′, and H-8′ of (−)-ampelopsin F were assigned *α*-, *β*-, *α*-, and *β*-orientations, respectively, whereas those of (+)-ampelopsin F were assigned *β*-, *α*-, *β*-, and *α*-orientations, respectively. The optical rotation of compound **2** $[\alpha {]}_{D}^{20}$: −64.9 (c 0.21 MeOH) is the same as that of (−)-Ampelopsin F $[\alpha {]}_{D}^{20}$: −4.56 (c 0.14 MeOH), and opposite to the optical rotation of compound (+)-Ampelopsin F $[\alpha {]}_{D}^{20}$: +14.00 (c 1.98 MeOH). It can be concluded that the relative configuration of H-7, 8, 7′, and 8′ is, respectively, *α*, *β*, *α*, and *β*. Finally, the experimental ECD curve of compound **2** showed good agreement with the calculated ECD curve (Figure 4), establishing its absolute configuration as 7S, 8S, 7′R, and 8′R. Based on the above analyses, compound **2** was identified as (−)-ampelopsin J, and the detailed data assignments are shown in Table 1.

### 2.2. Antioxidant Activities

Free radical scavenging is considered an important mechanism in the prevention of oxidative stress-related diseases. To evaluate the antioxidant potential of the isolated stilbene dimers, DPPH and ABTS^+^ radical scavenging assays were performed according to the method described in reference [25], with minor modifications. The scavenging activities of all compounds were determined at multiple concentrations, using ascorbic acid as the positive control. As shown in Table 2, stilbene dimers **1b** exhibited the strongest radical scavenging activity in both assays, with IC_50_ values of 14.86 ± 0.28 μg/mL for DPPH and 20.97 ± 1.70 μg/mL for ABTS^+^. These findings indicate that the hydroxyl substitution pattern critically influences antioxidant capacity.

### 2.3. α-Glucosidase-Inhibitory Activities

Inhibition of α-glucosidase is considered an effective strategy for controlling postprandial hyperglycemia and managing type 2 diabetes mellitus. To assess the *α*-glucosidase-inhibitory activities of the isolated stilbene dimers, the assay was conducted according to the method described in Ref. [26], with appropriate modifications. The inhibitory activities of all compounds were evaluated at different concentrations, with acarbose serving as the positive control. As summarized in Table 3, all three new stilbene dimers displayed potent *α*-glucosidase-inhibitory activity, with IC_50_ values ranging from 1.58 to 2.16 μg/mL, substantially lower than that of acarbose (IC_50_ = 227 μg/mL). These results indicate that the identified stilbene dimers have considerable potential as lead structures for the development of novel antidiabetic agents.

## 3. Discussion

The extremely low natural abundance of compounds **1a**, **1b**, and **2** constitutes a major limitation for their further biological evaluation and potential utilization. In the present study, only approximately 3 mg of the target compounds was obtained from 40 kg of fresh *H. patulum* fruits. Direct isolation from the plant material is therefore unlikely to provide sufficient quantities for comprehensive pharmacological studies, structure–activity relationship investigations, or preparative applications. Establishing how these compounds are formed may consequently be important not only for understanding their biosynthesis but also for developing alternative and more sustainable sources.

Based on their oligomeric stilbenoid frameworks, compounds **1a**, **1b**, and **2** may arise through oxidative coupling of hydroxylated stilbene precursors. The formation of naturally occurring resveratrol oligomers is generally proposed to involve oxidation of phenolic precursors to delocalized phenoxyl radicals, followed by regioselective C–C or C–O coupling and subsequent cyclization, rearrangement, or rearomatization reactions [27,28]. Such processes can generate considerable structural diversity because different radical-coupling positions and downstream transformations may produce distinct constitutional and stereochemical outcomes. Experimental studies provide precedent for this proposal. In particular, oxidation of piceatannol using Momordica charantia peroxidase afforded eight stilbene dimers, several of which exhibited *α*-glucosidase-inhibitory activity [29]. More recently, grapevine peroxidase VvPRX4 was shown to catalyze the conversion of trans-resveratrol and related stilbene substrates into dimers and higher oligomers, demonstrating that plant peroxidases can directly participate in stilbene oligomerization [30].

Nevertheless, these precedents do not establish the biosynthetic pathway of compounds **1a**, **1b**, and **2** in *H. patulum*. Their immediate precursor molecules remain unknown, and no enzyme from *H. patulum* has been demonstrated to catalyze their formation. It is also unclear which radical coupling positions generate the observed carbon skeletons, whether coupling occurs between identical or different stilbene precursors, and how regioselectivity and stereoselectivity are controlled. Accordingly, oxidative coupling should be regarded as a testable biosynthetic hypothesis rather than a confirmed pathway.

A systematic pathway elucidation strategy could combine comparative metabolomic and transcriptomic analyses with subsequent biochemical validation. Metabolite profiling of different fruit developmental stages or tissues may identify monomeric or dimeric stilbenes whose accumulation correlates with that of compounds **1a**, **1b**, and **2**. In parallel, transcriptomic analysis could be used to prioritize candidate oxidoreductases, particularly peroxidases, laccases, and related oxidative enzymes whose expression patterns correlate with oligostilbene accumulation [31]. Recombinant expression of these candidates, followed by in vitro incubation with the proposed precursors and LC–MS, NMR, and chiral analyses of the products, would be required to determine enzyme activity, substrate specificity, coupling regioselectivity, and stereochemical outcome.

Elucidation of the precursor–enzyme relationships could also provide a rational basis for overcoming material supply limitations. Potential approaches include elicited plant cell or tissue cultures, engineered microbial production of the required monomeric precursors, and subsequent enzyme-assisted or biomimetic oxidative coupling [27,30,31,32]. However, microbial production of stilbene monomers does not by itself ensure selective formation of the structurally complex oligomers. The principal challenge will be reproducing the regioselective and stereoselective coupling steps required for compounds **1a**, **1b**, and **2**. These production approaches should therefore be considered prospective strategies whose feasibility will depend on prior identification and functional characterization of the relevant precursors and oxidative enzymes.

Taken together, compounds **1a**, **1b**, and **2** exhibited distinct activity profiles in the two assay systems. Compound **1b** showed the strongest DPPH- and ABTS+-radical scavenging activities, whereas compound **1a** displayed the strongest *α*-glucosidase inhibition, with compound **2** showing intermediate activity in both evaluations. The different activity rankings observed in the antioxidant and enzyme inhibition assays indicate that these effects are unlikely to be governed by a single structural parameter, such as the number of phenolic hydroxyl groups. In particular, because compounds **1a** and **1b** are enantiomers with identical atomic connectivity and hydroxyl substitution patterns, their different activity profiles require consideration of stereochemical effects as well as possible assay-related factors. These findings prompted a broader examination of the relationships among stereochemistry, scaffold architecture, and biological activity in oligostilbenes.

Oligostilbenes represent a structurally diverse class of polyphenolic natural products, with their molecular architectures determined by multiple factors, including oxidative coupling patterns, degree of hydroxylation, central-ring frameworks, and stereochemical configurations. These structural variations may substantially influence their physicochemical properties and biological activities [33]. Previous studies on resveratrol-derived oligostilbenes, including viniferin-, ampelopsin-, and vaticanol-type compounds, have demonstrated that biological activities are not governed by a single structural feature but instead depend on the combined effects of oligomerization mode, molecular conformation, and functional-group arrangement [27,34,35].

Compounds **1a** and **1b** possess identical atomic connectivity and phenolic hydroxyl substitution patterns but opposite absolute configurations at the stereogenic centers within their rigid polycyclic cores and therefore constitute an enantiomeric pair. Accordingly, differences in their biological activities cannot be attributed to differences in molecular composition or hydroxyl group distribution. Because the DPPH and ABTS+ assays are conducted under essentially achiral chemical conditions, the stronger radical scavenging activity observed for **1b** should be interpreted cautiously and cannot be readily attributed to direct stereoselective recognition. Experimental variability, enantiomeric purity, compound stability, solubility, aggregation behavior, or other assay-related factors may have contributed to the observed difference.

In contrast, *α*-glucosidase is a chiral biological target, and the reproducibly stronger inhibition observed for **1a** may plausibly reflect stereoselective interactions with the enzyme active site. The three-dimensional orientation of the aromatic rings and phenolic hydroxyl groups in **1a** may provide a more favorable spatial arrangement for hydrogen bonding, aromatic interactions, and hydrophobic contacts, whereas the mirror-image configuration of **1b** may result in less favorable accommodation or greater steric constraints within the active site. However, this interpretation remains hypothetical and requires validation through enzyme kinetic studies, direct binding analyses, molecular modeling, structural investigations, and independent replication of the activity measurements.

Comparison with previously reported oligostilbenes further suggests that stereochemistry and scaffold architecture can influence biological properties. Viniferin-type oligostilbenes have shown differences in antioxidant and anti-inflammatory activities associated with their stereochemical configurations, although the magnitude and direction of these effects depend strongly on the assay system and biological context [34]. Similarly, vaticanol-type oligostilbenes have exhibited *α*-glucosidase-inhibitory activity, indicating that structurally distinct oligostilbene frameworks may contribute to enzyme inhibition [35]. However, the available data do not support a simple correlation between hydroxylation level or oligomer size and biological potency.

Compound **2** differs from compounds **1a** and **1b** not only in its hydroxylation pattern but also in the topology of its central scaffold. Therefore, activity differences involving compound **2** should be considered the combined consequences of molecular framework, coupling pattern, functional group arrangement, and three-dimensional presentation rather than being attributed solely to the number or positions of hydroxyl groups. Overall, these observations highlight the potential importance of stereochemical and conformational features in determining oligostilbene bioactivity, while emphasizing that further mechanistic studies are required to establish definitive structure–activity relationships.

## 4. Materials and Methods

### 4.1. General Experimental Procedure

HR-ESI-MS spectra were acquired using a Thermo Fisher Q Exactive-Plus spectrometer (Thermo Fisher Scientific, Waltham, MA, USA) with an electrospray ionization source (ESI). Ultra-high-performance liquid chromatography analyses were conducted on a Vanquish horizon spectrometer equipped with a Hypersil gold (2.12 × 250, 1.9 µm) column. NMR spectra were recorded using a BRUKER 600 NEO NMR spectrometer (Brock Co., Ltd., Karlsruhe, Germany). UV spectra were measured on a UV-2700 spectrometer (Shimadzu Co., Ltd., Tokyo, Japan). Optical rotation values were determined using an AUTOPOLVI spectrometer (Rudolph Co., Ltd., Hackettstown, NJ, USA). IR spectra were recorded on an IR Tracer-100 spectrometer (Shimadzu Co., Ltd., Kyoto, Japan). TLC was performed on silica gel GF254 plates (Qingdao Marine Chemical Ltd., Qingdao, China). Toyopearl HW-40F (Tosoh Corporation, Tokyo, Japan), Sephadex LH-20 (Pharmacia Biotech AB, Uppsala, Sweden) were used for column chromatography and Silica gel (Qingdao Marine Chemical Co., Ltd., Qingdao, China). 2,2-diphenyl-1-picrylhydrazyl (DPPH) (Shanghai McLean Biochemical Technology Co., Ltd., Shanghai, China), ascorbic acid (Shanghai McLean Biochemical Technology Co., Ltd., Shanghai, China), 2,2′-Azinobis-(3-ethylbenzthiazoline-6-sulphonate) (ABTS) (Sigma-Aldrich Co., Ltd., St. Louis, MO, USA), α-glucosidase (Sigma-Aldrich Co., Ltd., St. Louis, MO, USA), p-nitrophenyl-α-D-glucopyranoside (p-NPG) (Shanghai Yuanye Biotechnology Co., Ltd., Shanghai, China), acarbose (Bayer Health Care Ltd., Leverkusen, Germany).

### 4.2. Plant Material

*Hypericum patulum* Thunb. ex Murray (voucher specimen No. 20180801), a member of the Hypericaceae family, was collected in Guiyang, Guizhou Province, China (26°18′56′′ N, 106°46′9′′ E; elev. 1100 m) during July–August 2018. The material collected consisted of fresh ripe fruits. The species identification was confirmed by Professor Qingwen Sun of the Guizhou University of Traditional Chinese Medicine. The voucher specimen is deposited in the herbarium of the Guizhou Provincial Key Laboratory of Pharmaceutical Preparations, Guizhou Medical University.

### 4.3. Extraction and Isolation

The fruits of *H. patulum* (40 kg) were extracted three times with 30% ethanol (390 L total volume, *v*/*v*). The extract was concentrated under reduced pressure using a rotary evaporator, with the water bath temperature set at 50 °C, rotation speed at 80 rpm, and vacuum pressure maintained at approximately −0.08 MPa, yielding a crude extract of 3000 g. This extract was subsequently fractionated on a D101 macroporous adsorption resin column. Sequential elution was carried out with water (60 L, *v*/*v*) followed by 80% ethanol-water (180 L, *v*/*v*). The 80% ethanol–water eluate (1395 g) was further purified by silica gel CC. Elution employed a chloroform-methanol gradient (24 L, ranging from 10:0 to 6:4 *v*/*v*), yielding eleven combined fractions (F1–11). F9 was purified by normal-phase silica gel CC using an ethyl acetate–methanol gradient (9:1 → 5:5 *v*/*v*). Fractions exhibiting similar TLC profiles were combined and concentrated, yielding 9 sub-fractions (F9.1–9.9). F9.4 was fractionated on a Sephadex LH-20 column (methanol eluent). TLC-based pooling and concentration afforded 3 subfractions (F9.4.1–9.4.3). F9.4.3 was subjected to sequential chromatography: first on a normal-phase silica gel column (chloroform-methanol, 9:1 *v*/*v*), followed by a Sephadex LH-20 column (methanol). This process yielded compound **1** (7.1 mg). Compound **1** was subsequently resolved into a pair of enantiomers, **1a** (3.1 mg) and **1b** (3.5 mg), using a Chiral MX(2)-RH chiral column (250 mm × 4.6 mm, 5 µm) with isocratic elution (90% acetonitrile) (Supplementary Figure S11). F10 was fractionated on a Sephadex LH-20 column (methanol eluent). TLC-guided combination and concentration resulted in eleven subfractions (F10.1–10.11). F10.9 and F10.10 were merged and subjected to successive chromatography: normal-phase silica gel (petroleum ether–ethyl acetate, 6:4 *v*/*v*), Sephadex LH-20 (methanol), normal-phase silica gel (chloroform–acetone, 1:1 *v*/*v*), and finally normal-phase silica gel (dichloromethane–methanol, 9:1 *v*/*v*), yielding compound **2** (89.4 mg).

(+)-caesalstilbene C (**1a**): Brown solid; $[\alpha {]}_{D}^{20}$: +32.33 (*c* 0.1, MeOH); UV (MeOH) λmax (log ε) 284 (4.23) nm; IR (KBr) *ν*max 3325, 2911, 1604, 1511, 1460, 1260, 1286, 1157 cm^−1^, ^1^H NMR and ^13^C NMR data, see Table 1; HR-ESI-MS *m*/*z*: 501.1160 [M − H]^−^, calculated for C_28_H_21_O_9_ 501.1180 (Supplementary Figure S1).

(−)-caesalstilbene C (**1b**): Brown solid; $[\alpha {]}_{D}^{20}$: −30.67 (*c* 0.1, MeOH); UV (MeOH) *λ*max (log *ε*) 284 (4.23) nm; IR (KBr) *ν*max 3325, 2911, 1604, 1511, 1460, 1260, 1286, 1157 cm^−1^, ^1^H NMR and ^13^C NMR data, see Table 1; HR-ESI-MS *m*/*z*: 501.1160 [M−H]^−^, calculated for C_28_H_21_O_9_ 501.1180.

(−)-Ampelopsin J (**2**): Pale yellow solid; $[\alpha {]}_{D}^{20}$: −64.9 (c 0.21, MeOH); UV (MeOH) *λ*max (log ε) 285 (4.05) nm; IR (KBr) *ν*max 3339, 2931, 1606, 1518, 1465, 1253, 1146, 1114 cm^−1^, ^1^H NMR and ^13^C NMR data, see Table 1; HR-ESI-MS *m*/*z*: 485.1244 [M − H]^−^, calculated for C_28_H_21_O_8_ 485.1231 (Supplementary Figure S12).

### 4.4. Electronic Circular Dichroism Calculation of Compounds ***1***–***2***

Conformational analysis via random searching (Sybyl-X 2.0, MMFF94S force field, 5 kcal/mol cutoff) identified six low-energy conformers. Subsequent geometry optimization and frequency analysis at the B3LYP-D3(BJ)/6-31G*//CPCM(methanol) level (ORCA 5.0.1) confirmed all structures as stable minima (no imaginary frequencies). Time-dependent density functional theory (TD-DFT) calculations at the PBE0/def2-TZVP//CPCM(methanol) level (ORCA 5.0.1) computed excitation energies, oscillator strengths, and rotational strengths (velocity) for the first 60 excited states. Electronic circular dichroism (ECD) spectra were simulated using Gaussian functions (σ = 0.30 eV). Boltzmann-averaged spectra, weighted by relative Gibbs free energies (ΔG) derived from B3LYP-D3(BJ)/6-31G* thermal corrections and wB97M-V/def2-TZVP electronic energies, were compared to experimental data.

### 4.5. Antioxidant Activities

#### 4.5.1. DPPH Radical Scavenging Activities

The DPPH radical scavenging assay has been suitably modified as said by the methodology described in the literature [25]. Briefly, in a 96-well microplate, the following mixtures were prepared: (1) Test Sample (A_1_); (2) Sample Background Control (A_2_); (3) DPPH Control (A_3_). All samples were tested at seven graded concentrations ranging from 0.1 to 1000 μg/mL, with each concentration tested in triplicate wells, and the entire experiment was independently repeated three times. All wells were mixed gently and incubated in the dark at room temperature for 30 min. Absorbance at 517 nm was then measured using a microplate reader. Ascorbic acid solution was used as a positive control, following an identical procedure. The DPPH radical scavenging activity was calculated using the following equation: DPPH radical scavenging activity (%) = [1 − (A_1_ − A_2_)/A_3_] × 100%.

The half-maximal inhibitory concentration (IC_50_) values were calculated by nonlinear regression analysis using a four-parameter logistic curve fitting of inhibition percentage against the logarithm of sample concentrations, performed with GraphPad Prism 8.0 (GraphPad Software, San Diego, CA, USA). All data are presented as the mean ± standard deviation (SD) from three independent experiments.

#### 4.5.2. ABTS+ Radical Scavenging Activities

The ABTS^+^ radical cation scavenging assay was performed using the same procedure as described in Section 4.5.1 for the DPPH assay, with the following modifications: the DPPH reagent was replaced with ABTS^+^ radical cation solution; absorbance was measured at 734 nm instead of 517 nm; and the reaction mixtures were prepared as test measurement (Ai), sample background control (Aj), and reagent blank (Ac). The same concentration range (0.1–1000 μg/mL), replicate numbers (triplicate wells per concentration, three independent repeats), positive control (ascorbic acid), incubation conditions (30 min in the dark at room temperature), and IC_50_ calculation method (nonlinear regression with GraphPad Prism 8.0) were applied as in Section 4.5.1. The ABTS^+^ radical cation scavenging activity was calculated using the following equation: ABTS^+^ radical cation scavenging activity (%) = [1 − (Ai − Aj)/Ac] × 100%.

All data are expressed as the mean ± SD from three independent experiments.

### 4.6. The Inhibitory Activity Towards α-Glucosidase

The α-glucosidase-inhibitory activity was evaluated according to a previously reported method [26], with appropriate modifications. Briefly, the sample solution, phosphate-buffered saline and α-glucosidase solution were added to each well of a microplate, mixed thoroughly, and incubated at 37 °C for 15 min. Subsequently, a solution of p-nitrophenyl-α-D-glucopyranoside (p-NPG) was added as the substrate. The reaction mixture was mixed thoroughly and incubated at 37 °C for an additional 25 min. After the reaction was terminated, the absorbance at 405 nm was measured immediately using a microplate reader.

All data are presented as the mean ± SD from three independent experiments performed in triplicate. Statistical analysis was performed using one-way ANOVA with Tukey’s post-hoc test, with *p* < 0.05 considered significant; 95% confidence intervals were calculated for IC_50_ values where applicable. GraphPad Prism 8.0 was used for all calculations.

## 5. Conclusions

Three stilbene dimers, (+)-caesalstilbene C (**1a**), (−)-caesalstilbene C (**1b**), and (−)-ampelopsin J (**2**), were isolated from the 30% ethanol extract of the fruits of *Hypericum patulum*. Compounds **1a** and **1b** were separated by chiral HPLC in an approximately 1:1 ratio, and their absolute configurations were established by comparison of their experimental and calculated ECD spectra. In the antioxidant assays, compound **1b** exhibited the strongest DPPH and ABTS radical scavenging activities, with IC_50_ values of 14.86 ± 0.28 and 20.97 ± 1.70 μg/mL, respectively. In addition, compounds **1a**, **1b**, and **2** inhibited α-glucosidase, with IC_50_ values ranging from 1.58 to 2.16 μg/mL under the present assay conditions.

These findings expand the known chemical diversity of *H. patulum* fruits and provide preliminary evidence for the in vitro radical scavenging and α-glucosidase-inhibitory activities of these stilbene dimers. However, these assays alone do not establish antioxidant efficacy in biological systems, antidiabetic activity, safety, bioavailability, or therapeutic potential. In addition, the extremely low natural abundance of the compounds limits their further pharmacological evaluation by direct isolation. Future studies should therefore verify the observed activities through mechanistic, cellular, and in vivo investigations and develop practical alternative approaches for obtaining sufficient quantities of these compounds.

## Figures and Tables

**Table 1:** ^1^H NMR (600 MHz) and ^13^C NMR (150 MHz) data (*J* in Hz, in CD_3_OD) of **1**–**2**.

No.	1		2	
	*δ*_H_	*δ*_C_	*δ*_H_	*δ*_C_
1		140.3		140.0
2	6.49 (d, 2.4)	115.3	6.64 (d, 2.1)	116.5
3		146.2		145.7
4		144.4		144.1
5	6.68 (d, 8.6)	116.2	6.71 (d, 8.1)	116.1
6	6.50 (dd, 8.6, 2.4)	119.6	6.57 (dd, 8.1, 2.1)	120.6
7	4.21 (s)	58.9	4.02 (s)	47.8
8	3.81 (d, 7.7)	61.0	4.02 (s)	49.6
9		150.2		114.2
10		123.3		158.1
11		155.7	6.08 (d, 2.3)	101.9
12	6.45 (d, 2.0)	103.2		157.1
13		159.3	6.34 (d, 2.3)	105.7
14	6.12 (d, 2.0)	102.5		147.9
1′		138.0		137.0
2′		159.2	6.33 (d, 2.1)	115.9
3′		159.2		145.6
4′	6.08 (d, 2.3)	101.0		144.0
5′		159.2	6.49 (d, 8.2)	115.7
6′	6.14 (d, 2.3)	106.9	6.23 (dd, 8.2, 2.1)	119.8
7′	4.33 (s)	56.0	3.56 (s)	51.2
8′	3.83 (d, 7.7)	60.7	3.22 (s)	59.5
9′		151.3		147.6
10′		138.6	6.36 (d, 2.0)	104.3
11′	6.42 (s)	112.7		153.1
12′		146.0	6.01 (d, 2.0)	101.9
13′		146.0		158.4
14′	6.93 (s)	111.4		128.8

**Table 2:** DPPH and ABTS^+^ scavenging activities of compounds **1**–**2**.

Compound	DPPH IC_50_ (μg/mL)	ABTS IC_50_ (μg/mL)
**1a**	22.02 ± 0.29	32.49 ± 1.20
**1b**	14.86 ± 0.28	20.97 ± 1.70
**2**	18.12 ± 0.39	26.21 ± 0.62
**ascorbic acid**	20.41 ± 0.06	46.21 ± 0.28

Note: compared with the positive control (ascorbic acid) group.

**Table 3:** The inhibitory activity towards *α*-glucosidase **1**–**2**.

Compound	IC_50_ (μg/mL)
**1a**	1.58 ± 0.08
**1b**	2.16 ± 0.11
**2**	1.75 ± 0.04
**acarbose**	227.53 ± 10.56

Note: compared with the positive control (acarbose) group.

## Data Availability

The data underlying this article are available in the article and Supplementary Materials.

## Supplementary Materials

The following supporting information can be downloaded at: https://www.mdpi.com/article/10.3390/molecules31173072/s1, Figure S1. HR-ESI-MS spectrum of compound **1**. Figure S2. UV spectrum of compound **1**. Figure S3. IR spectrum of compound **1**. Figure S4. ^1^H-NMR (600 MHz, CD^3^OD) spectrum of compound **1**. Figure S5. ^13^C-NMR (150 MHz, CD^3^OD) spectrum of compound **1**. Figure S6. HMQC spectrum of compound **1**. Figure S7. HMBC spectrum of compound **1**. Figure S8. NOESY spectrum of compound **1**. Figure S9. ^1^H-^1^HCOSY spectrum of compound **1**. Figure S10. CD spectrum of compound **1**. Figure S11. Chiral HPLC chromatograms of compound **1** obtained using a Chiral MX(2)-RH column (250 mm × 4.6 mm, 5 μm) under isocratic elution with 90% acetonitrile as the mobile phase and detection at 245 and 220 nm. Figure S12. HR-ESI-MS and MS/MS spectra of compound **2**. Figure S13. UV spectrum of compound **2**. Figure S14. IR spectrum of compound **2**. Figure S15. ^1^H-NMR (600 MHz, CD^3^OD) spectrum of compound **2**. Figure S16. ^13^C-NMR (150 MHz, CD^3^OD) spectrum of compound **2**. Figure S17. HMQC spectrum of compound **2**. Figure S18. HMBC spectrum of compound **2**. Figure S19. NOESY spectrum of compound **2**. Figure S20. ^1^H-^1^HCOSY spectrum of compound **2**. Figure S21. CD spectrum of compound **2**.
