# Supplementary material for: New Stilbene Dimer Compounds from the Fruits of Hypericum patulum and Their Antioxidant and Anti-α-Glucosidase Activities
Source: Molecules. 2026 Sep 1;31(17):3072. doi: 10.3390/molecules31173072 (PMC13567735; doi:10.3390/molecules31173072)
Supplement: Supplementary file 1 [file molecules-31-03072-s001.zip › molecules-4451177-supplementary.pdf]

Contents

Figure S1. HR-ESI-MS spectrum of compound **1**.

Figure S2. UV spectrum of compound **1**.

Figure S3. IR spectrum of compound **1**.

Figure S4. ^{1}H-NMR (600MHz, CD_3OD) spectrum of compound **1**.

Figure S5. ^{13}C-NMR (150MHz, CD_3OD) spectrum of compound **1**.

Figure S6. HMQC spectrum of compound **1**.

Figure S7. HMBC spectrum of compound **1**.

Figure S8. NOESY spectrum of compound **1**.

Figure S9. ^{1}H-^{1}HCOSY spectrum of compound **1**.

Figure S10. CD spectrum of compound **1**.

Figure S11. Chiral HPLC chromatograms of compound 1 obtained using a Chiral MX(2)-RH column (250 mm × 4.6 mm, 5 μm) under isocratic elution with 90% acetonitrile as the mobile phase and detection at 245 and 220 nm.

Figure S12. HR-ESI-MS and MS/MS spectra of compound **2**.

Figure S13. UV spectrum of compound **2**.

Figure S14. IR spectrum of compound **2**.

Figure S15. ^{1}H-NMR (600MHz, CD_3OD) spectrum of compound **2**.

Figure S16. ^{13}C-NMR (150MHz, CD_3OD) spectrum of compound **2**.

Figure S17. HMQC spectrum of compound **2**.

Figure S18. HMBC spectrum of compound **2**.

Figure S19. NOESY spectrum of compound **2**.

Figure S20. ^{1}H-^{1}HCOSY spectrum of compound **2**.

Figure S21. CD spectrum of compound **2**.

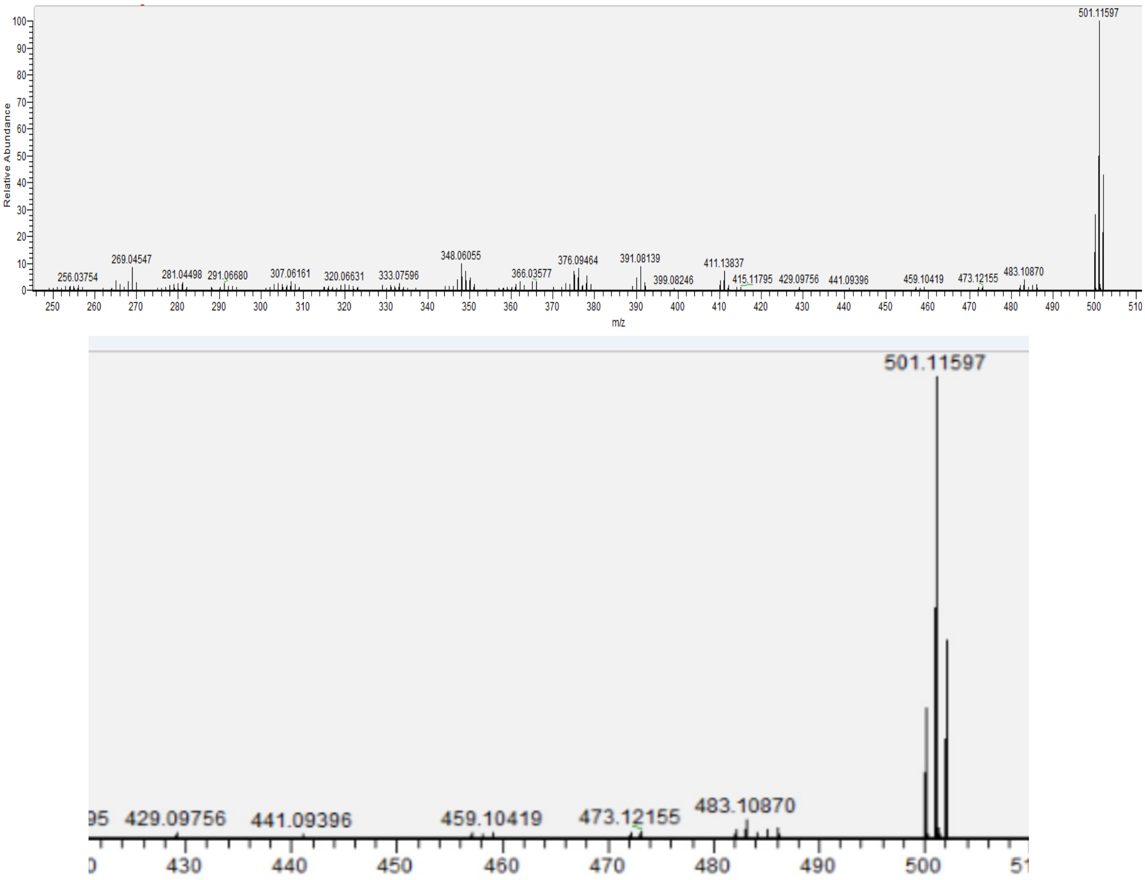

Figure S1. HR-ESI-MS spectrum of compound **1**.

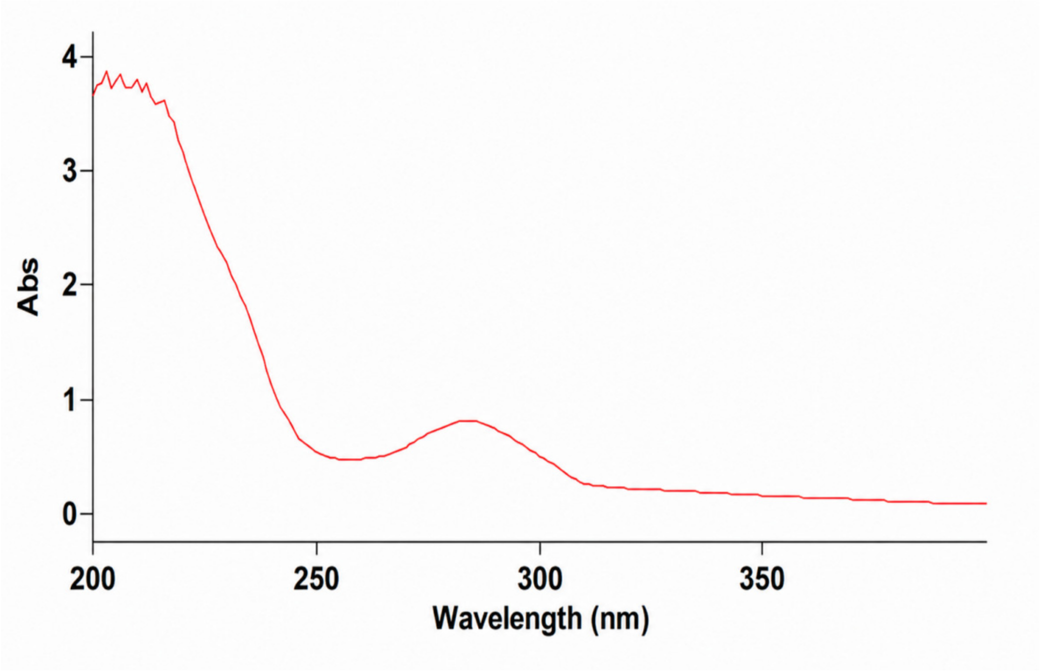

Figure S2. UV spectrum of compound **1**.

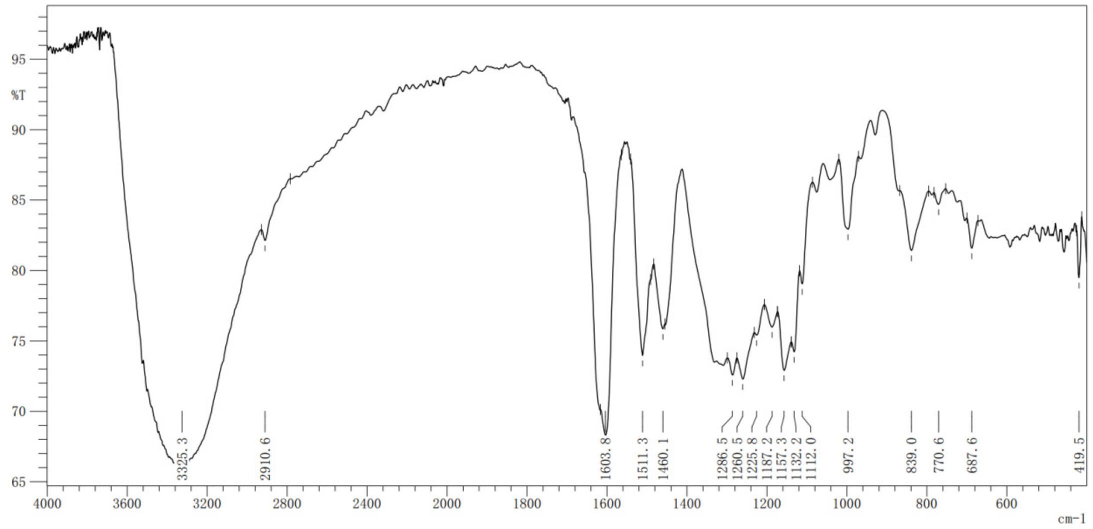

Figure S3. IR spectrum of compound **1**.

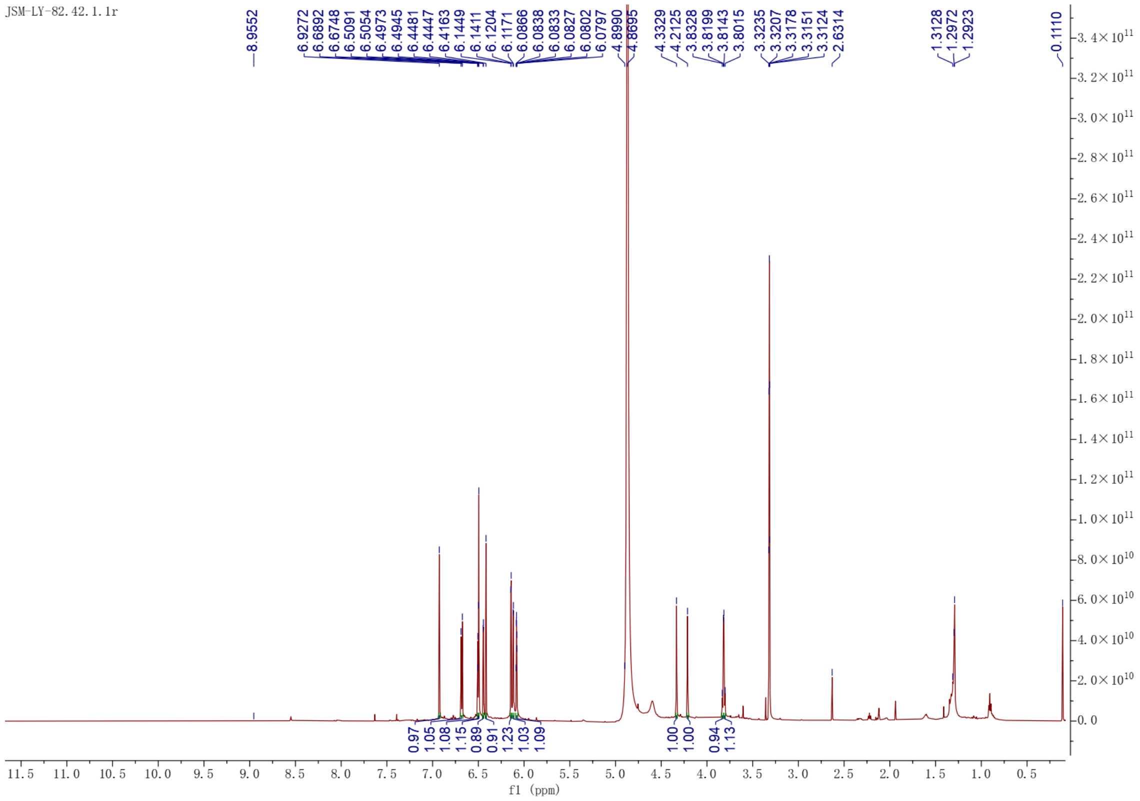

Figure S4. ^{1}H-NMR (600MHz, CD_3OD) spectrum of compound **1**.

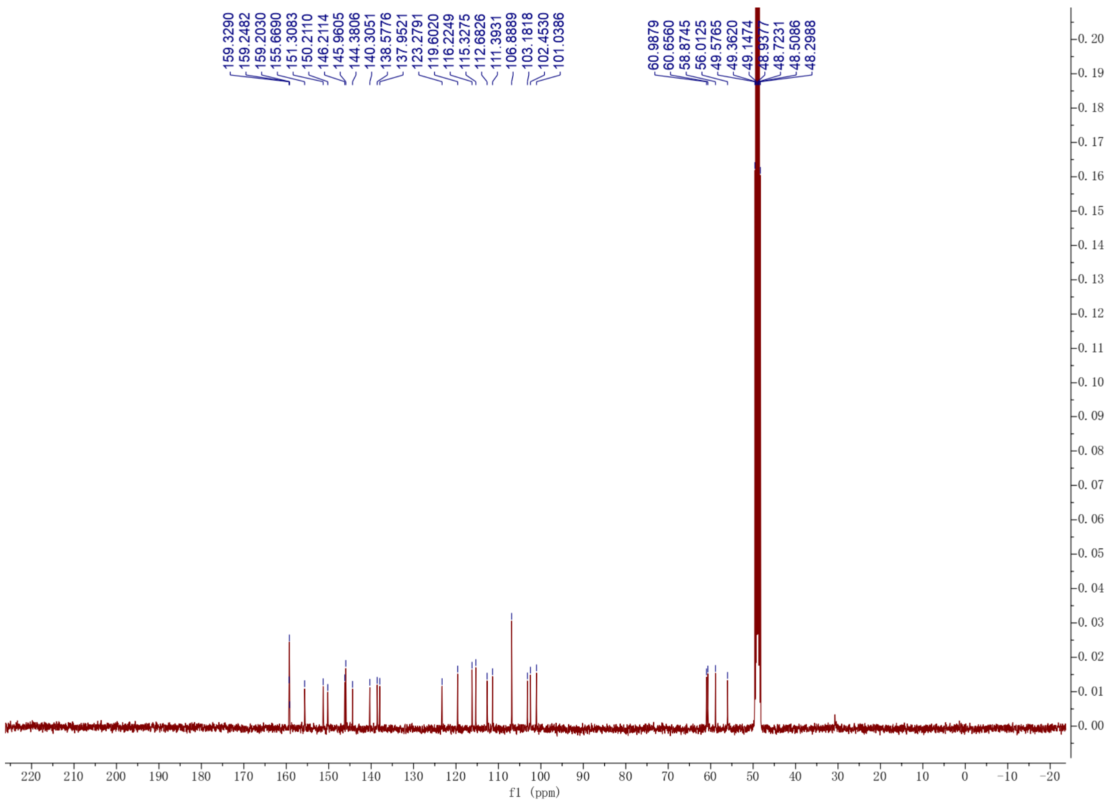

Figure S5. ^{13}C-NMR (150MHz, CD_3OD) spectrum of compound **1**.

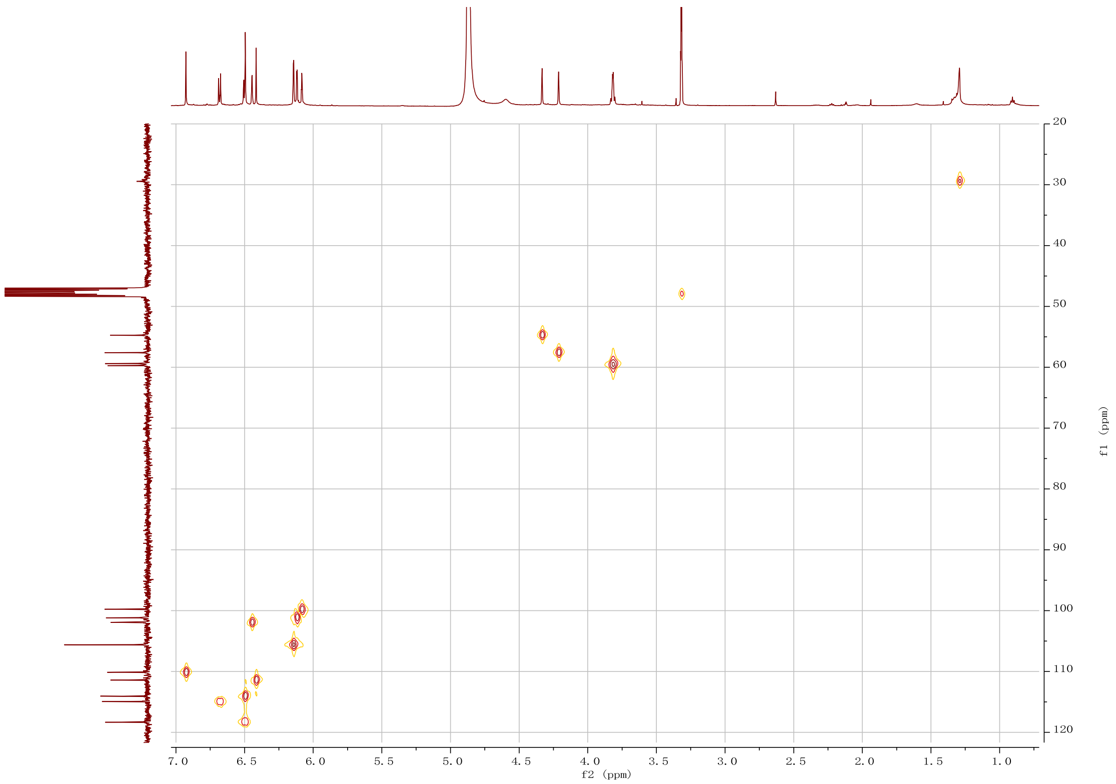

Figure S6. HMQC spectrum of compound **1**.

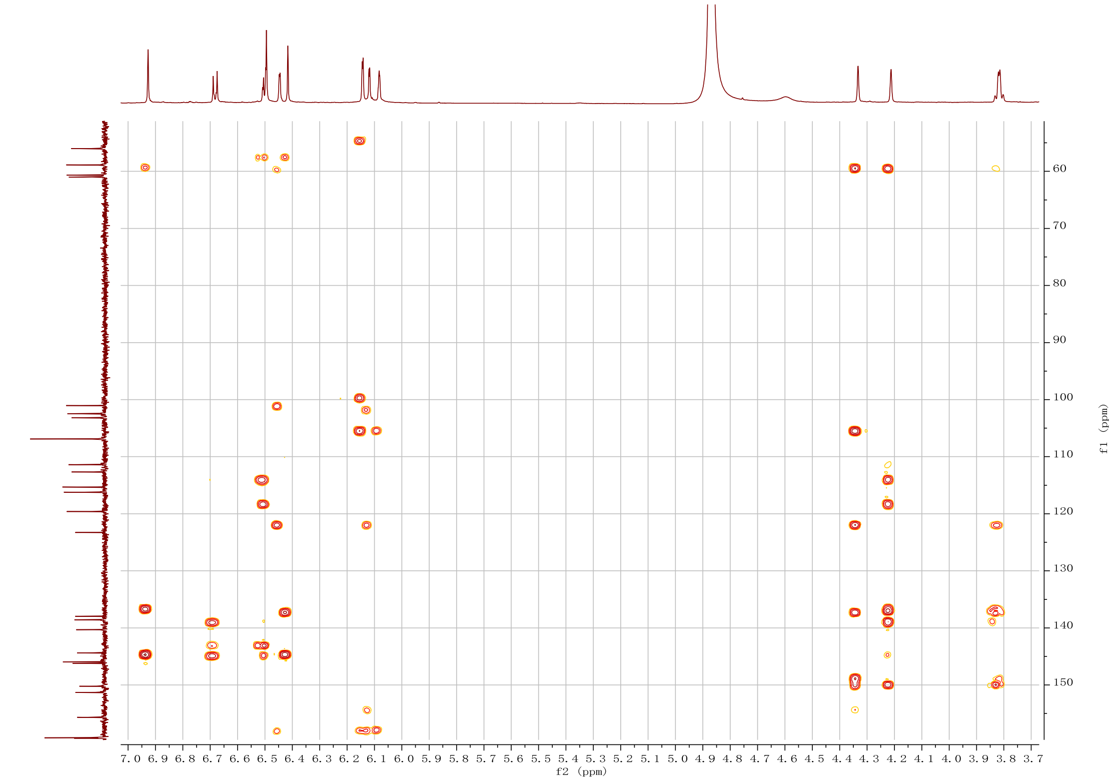

Figure S7. HMBC spectrum of compound **1**.

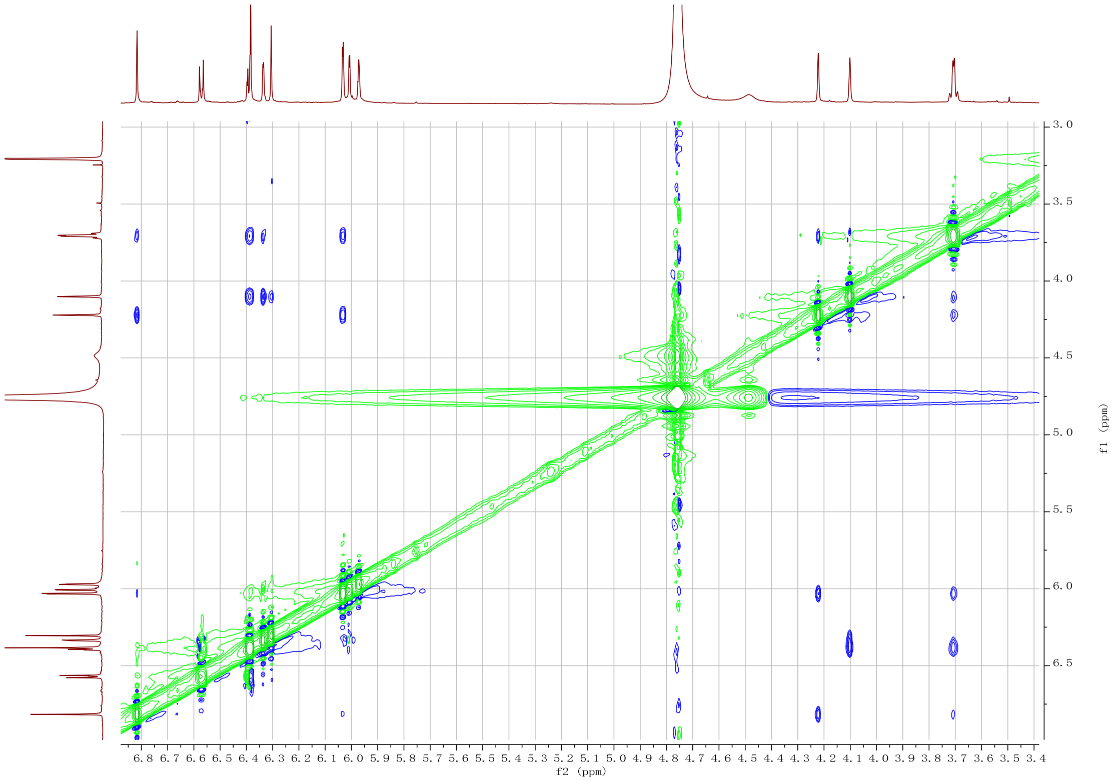

Figure S8. NOESY spectrum of compound **1**.

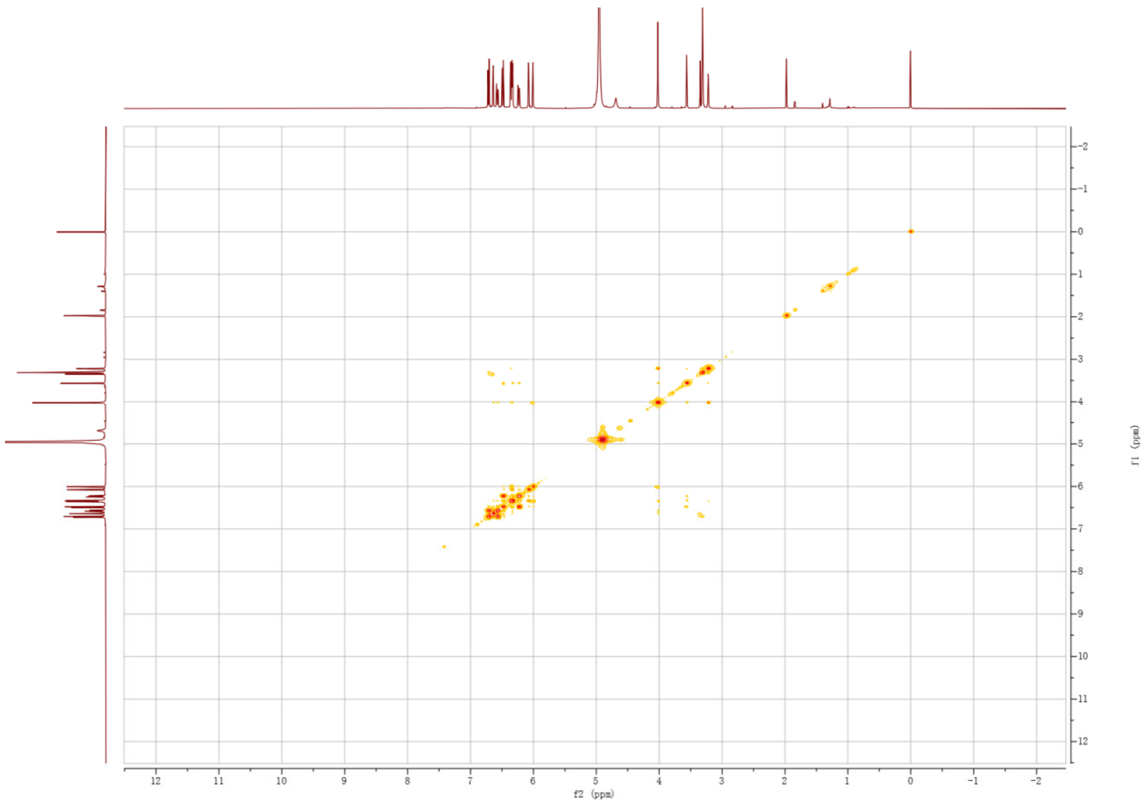

Figure S9. ^{1}H-^{1}HCOSY spectrum of compound **1**.

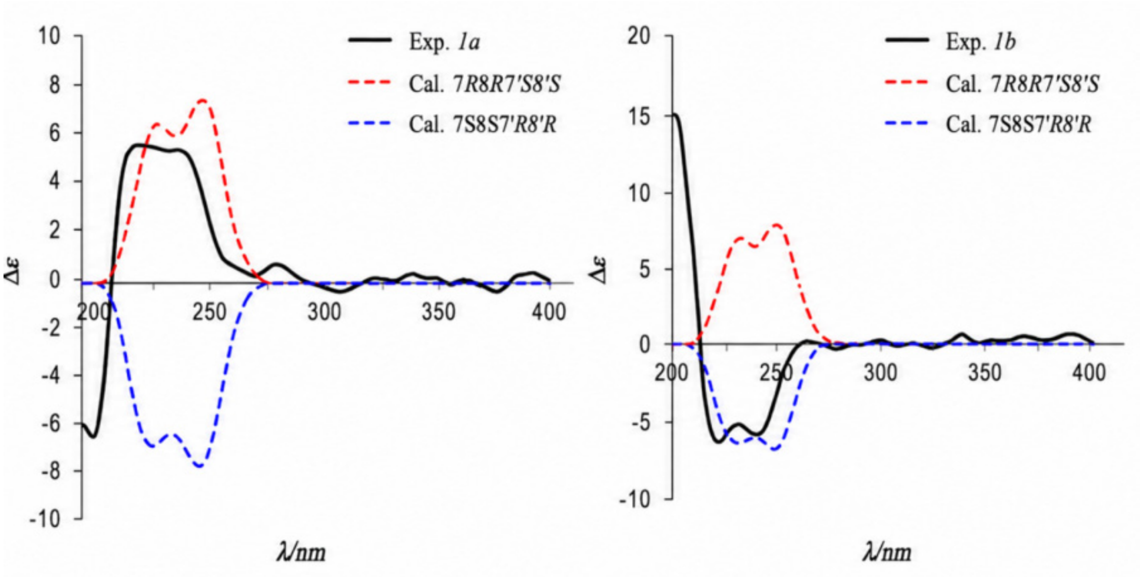

Figure S10. CD spectrum of compound **1**.

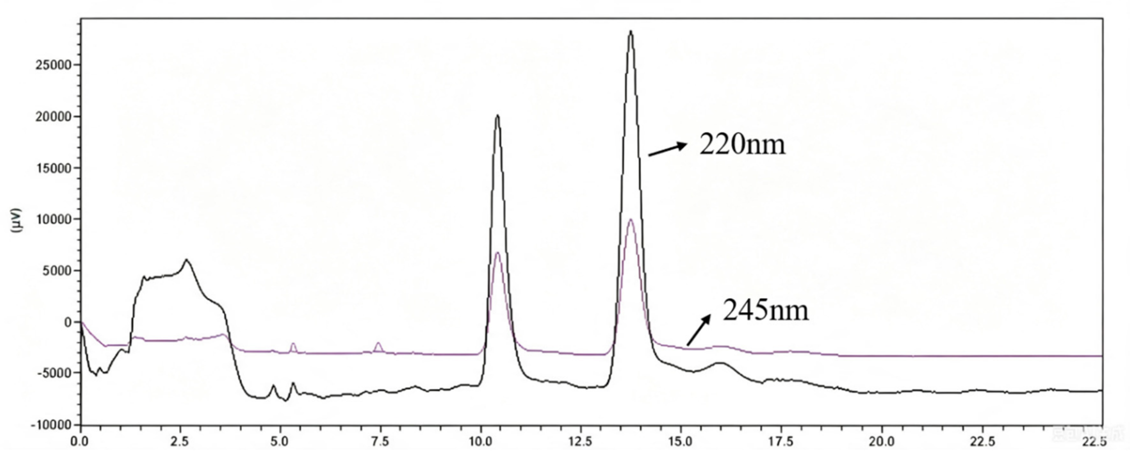

Figure S11. Chiral HPLC chromatograms of compound 1 obtained using a Chiral MX(2)-RH column (250 mm × 4.6 mm, 5 μm) under isocratic elution with 90% acetonitrile as the mobile phase and detection at 245 and 220 nm.

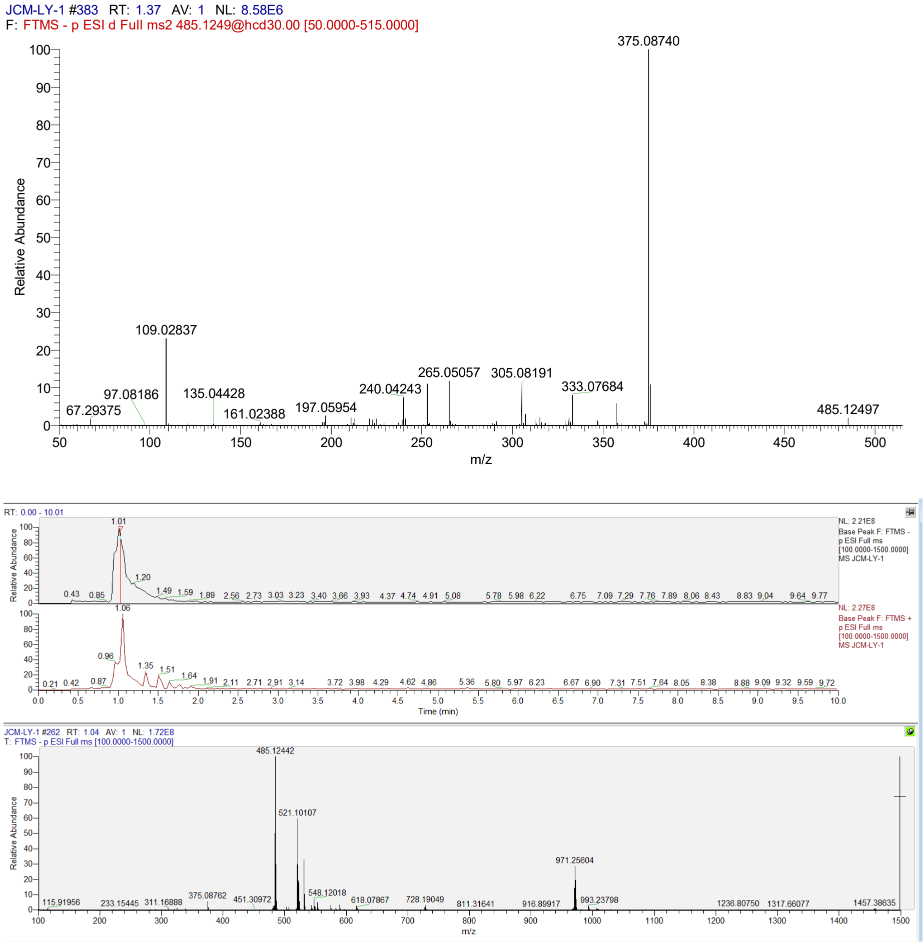

Figure S12. HR-ESI-MS and MS/MS spectra of compound **2**.

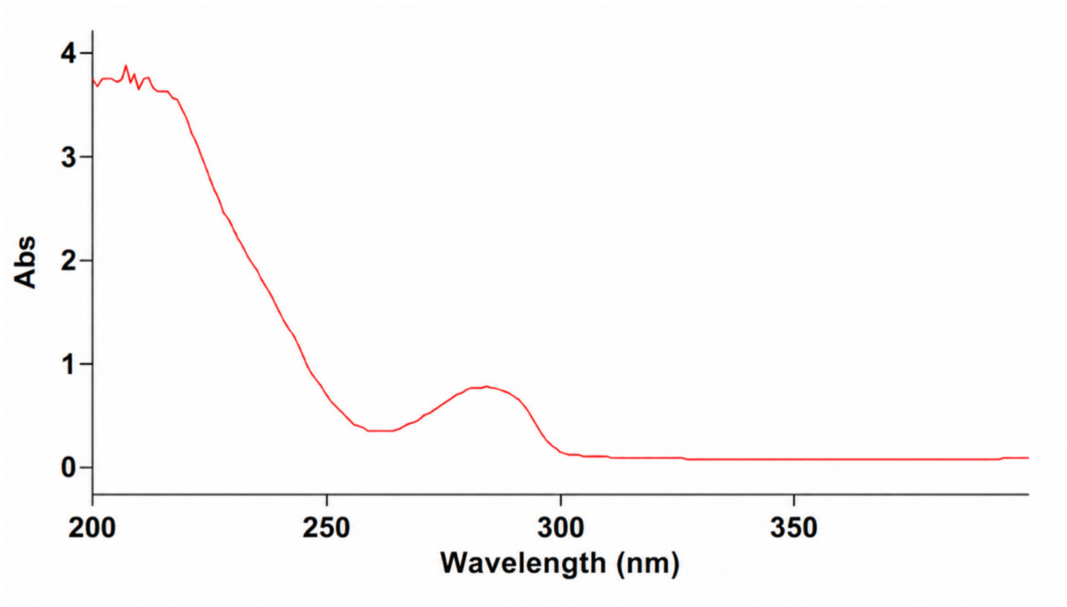

Figure S13. UV spectrum of compound **2**.

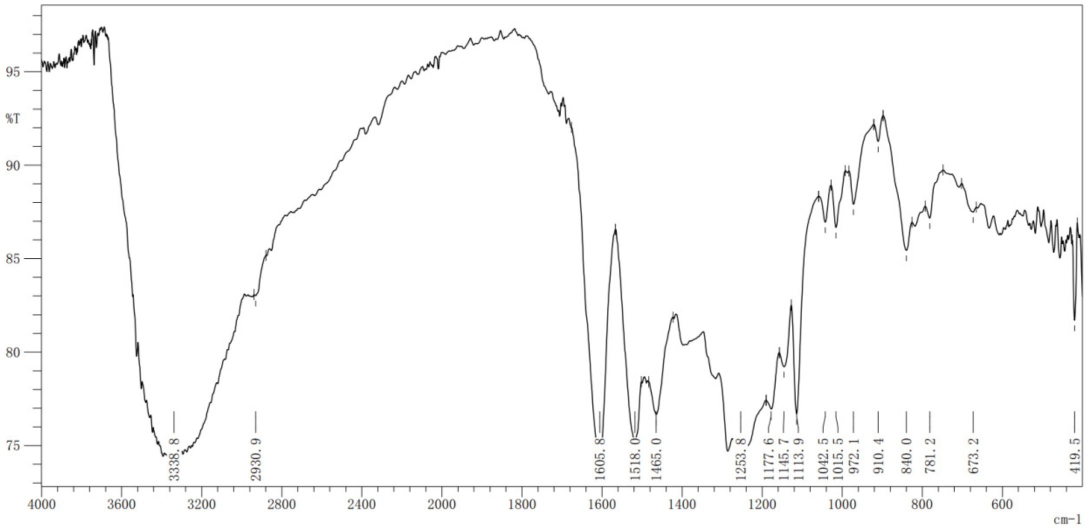

Figure S14. IR spectrum of compound **2**.

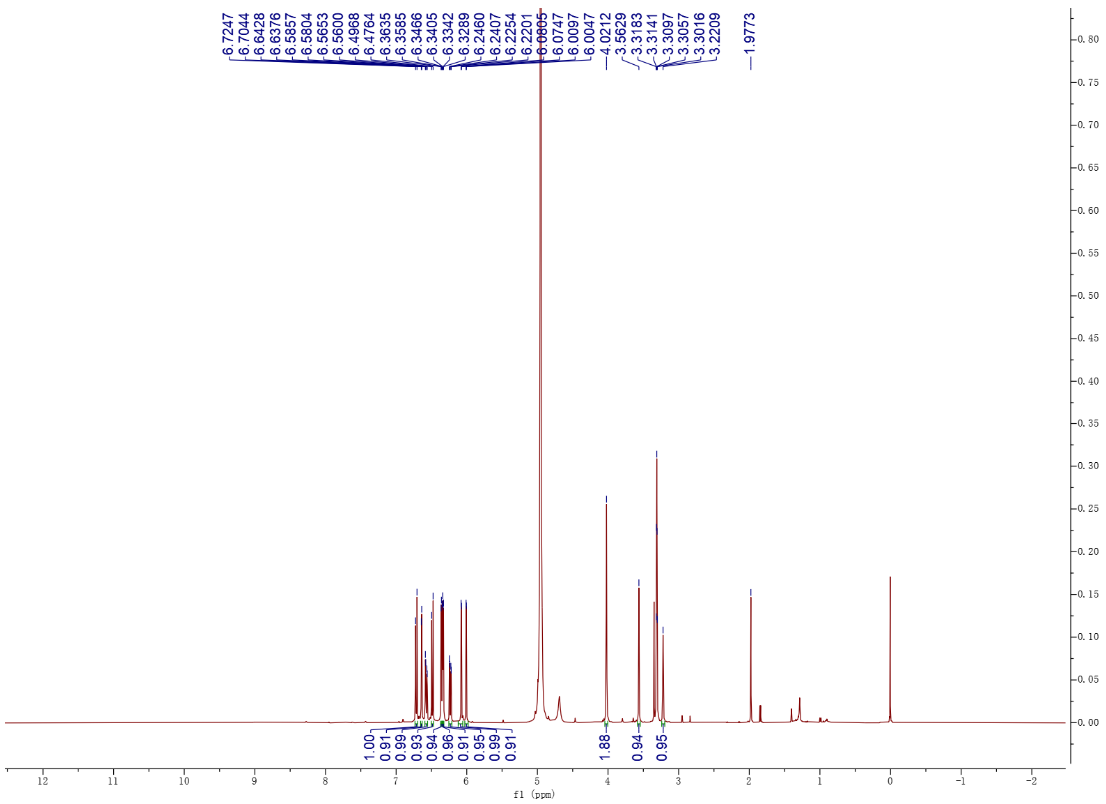

Figure S15. ^{1}H-NMR (600MHz, CD_3OD) spectrum of compound **2**.

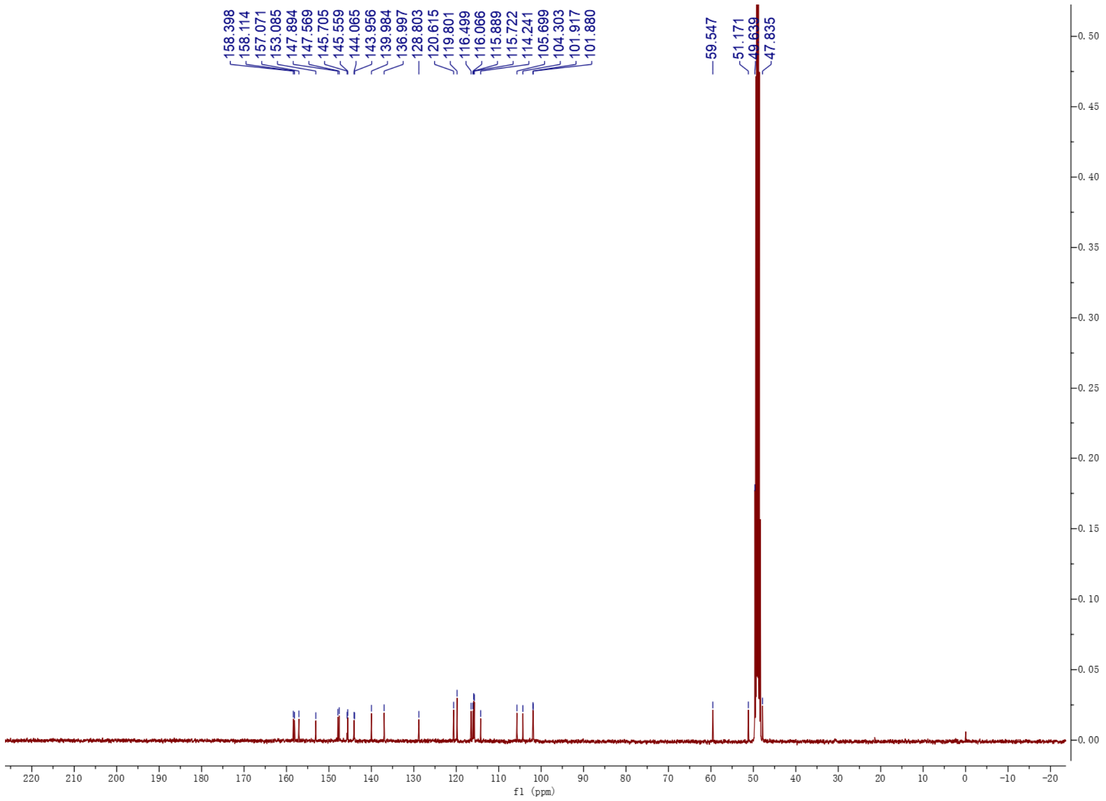

Figure S16. ^{13}C-NMR (150MHz, CD_3OD) spectrum of compound **2**.

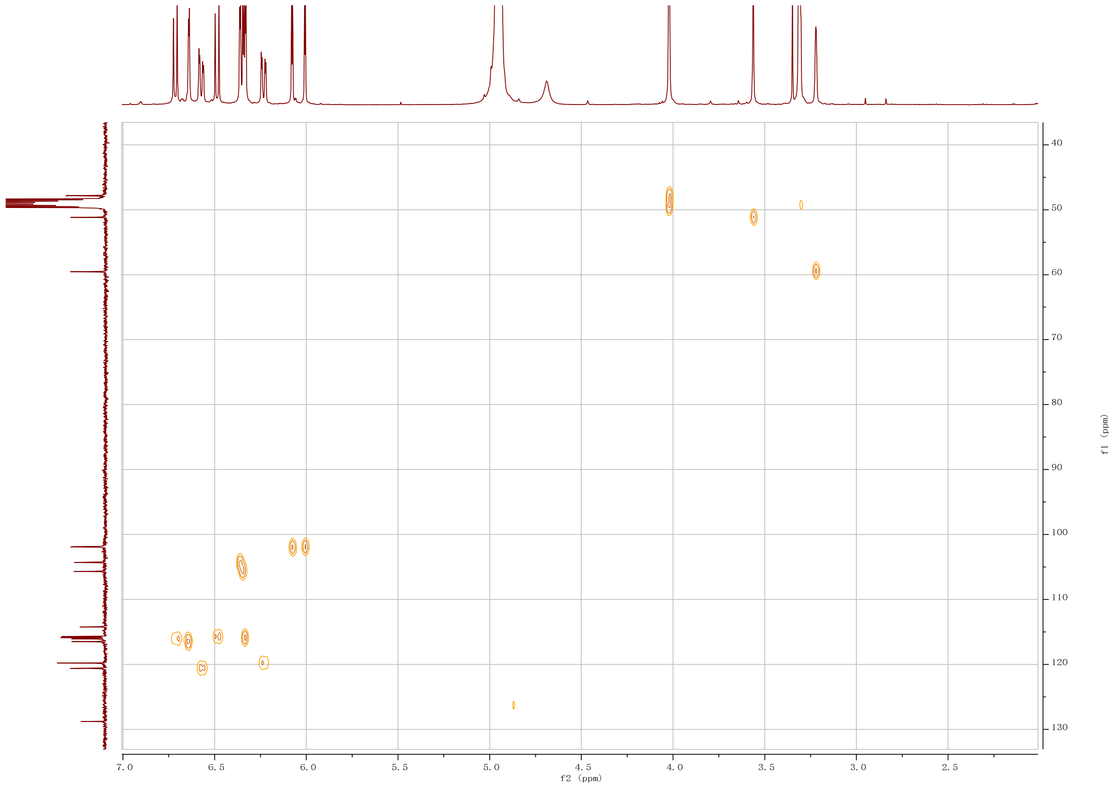

Figure S17. HMQC spectrum of compound **2**.

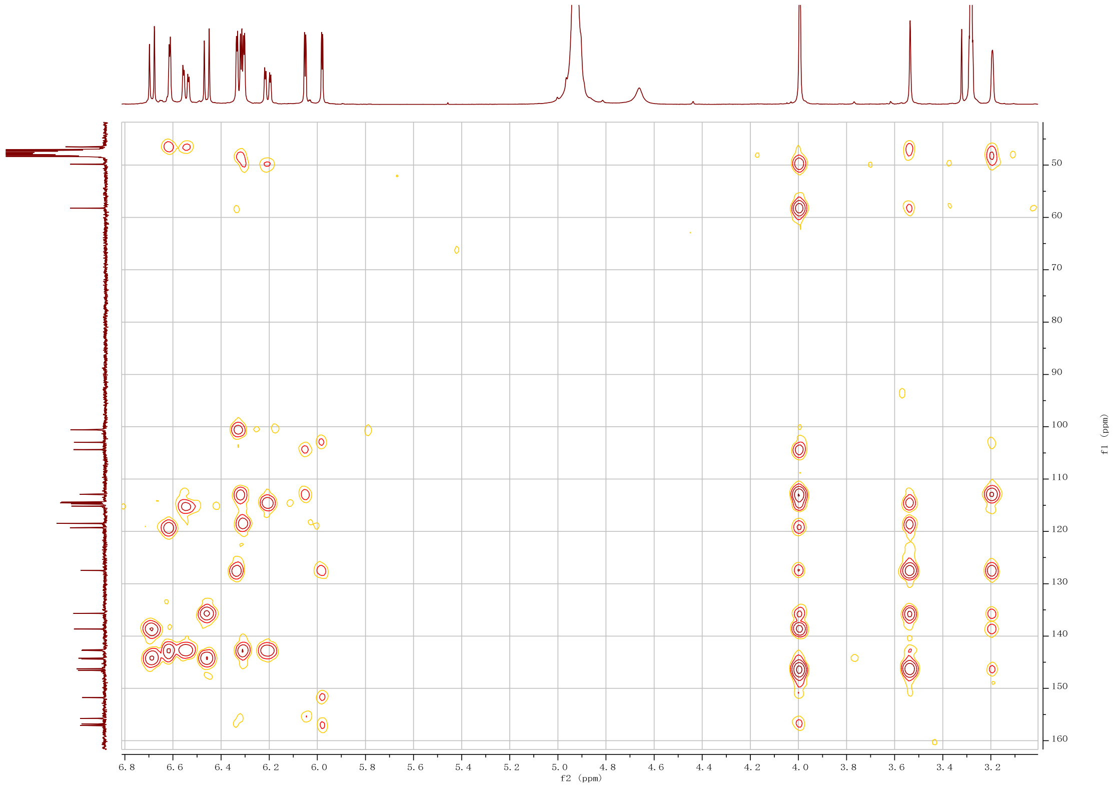

Figure S18. HMBC spectrum of compound **2**.

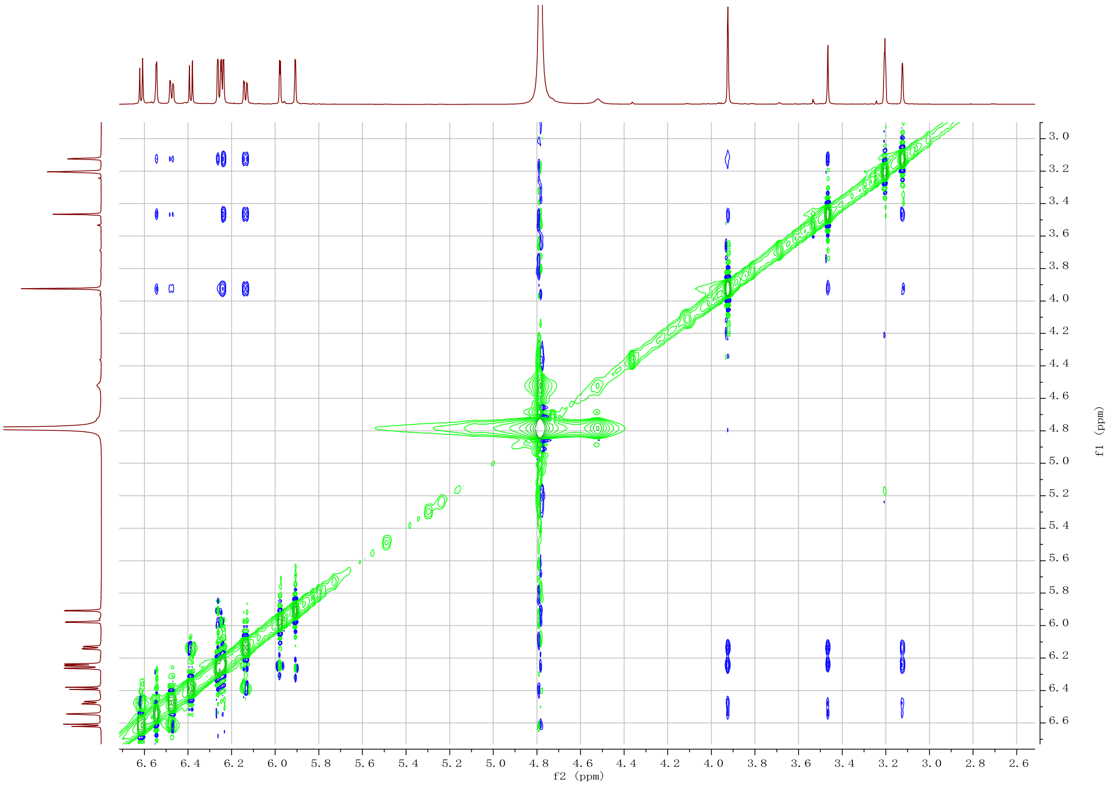

Figure S19. NOESY spectrum of compound **2**.

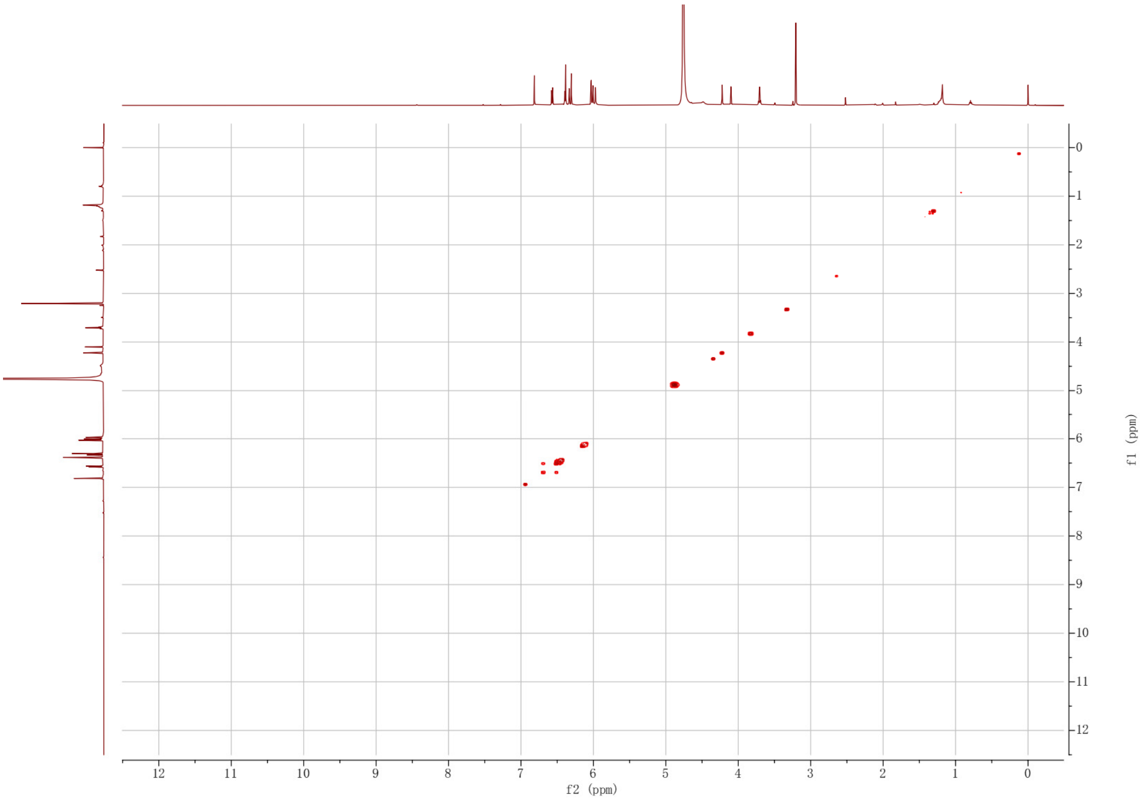

Figure S20. ^{1}H-^{1}HCOSY spectrum of compound **2**.

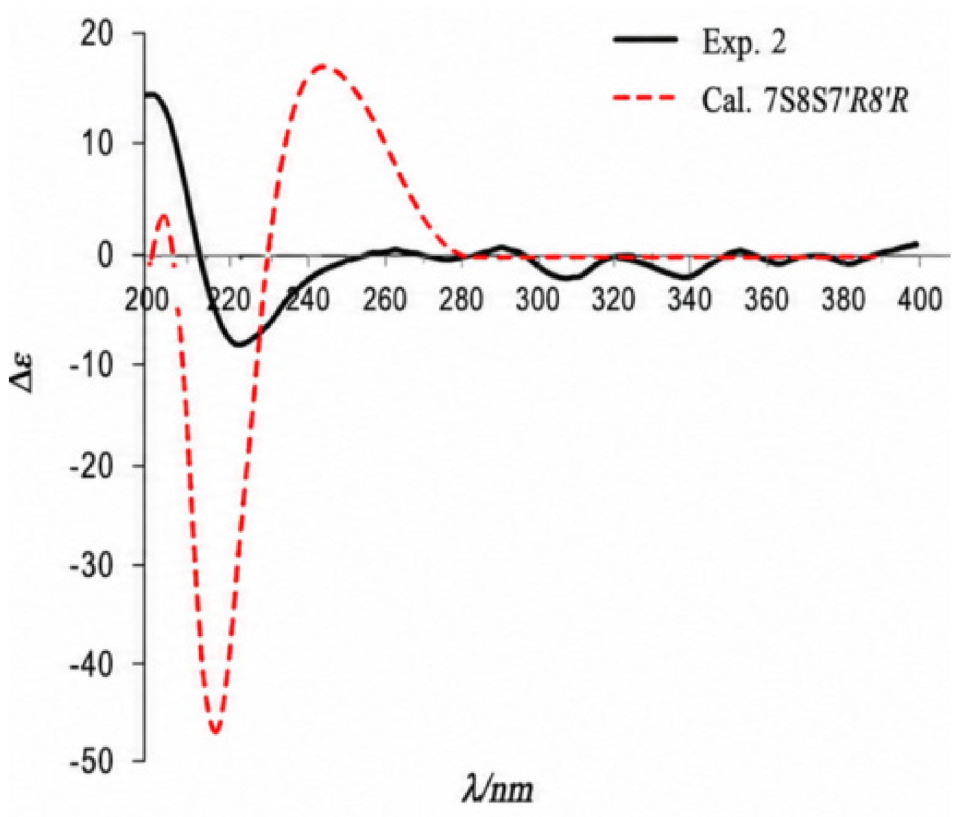

Figure S21. CD spectrum of compound **2**.
